# Thermoresponsive Poly(glycidyl ether) Brush Coatings on Various Tissue Culture Substrates—How Block Copolymer Design and Substrate Material Govern Self-Assembly and Phase Transition

**DOI:** 10.3390/polym12091899

**Published:** 2020-08-24

**Authors:** Daniel David Stöbener, Marie Weinhart

**Affiliations:** 1 Institute of Physical Chemistry and Electrochemistry, Leibniz Universität Hannover, Callinstr. 3A, 30167 Hannover, Germany; daniel.stoebener@pci.uni-hannover.de; 2 Institute of Chemistry and Biochemistry, Freie Universität Berlin, Takustr. 3, 14195 Berlin, Germany

**Keywords:** critical aggregation concentration, coil-to-globule transition, mesoglobules, thermal hysteresis, physical adsorption, C,H-insertion crosslinking, grafting density, brush conformation, volume phase transition, temperature-dependent wettability

## Abstract

Thermoresponsive poly(glycidyl ether) brushes can be grafted to applied tissue culture substrates and used for the fabrication of primary human cell sheets. The self-assembly of such brushes is achieved via the directed physical adsorption and subsequent UV immobilization of block copolymers equipped with a short, photo-reactive benzophenone-based anchor block. Depending on the chemistry and hydrophobicity of the benzophenone anchor, we demonstrate that such block copolymers exhibit distinct thermoresponsive properties and aggregation behaviors in water. Independent on the block copolymer composition, we developed a versatile grafting-to process which allows the fabrication of poly(glycidyl ether) brushes on various tissue culture substrates from dilute aqueous-ethanolic solution. The viability of this process crucially depends on the chemistry and hydrophobicity of, both, benzophenone-based anchor block and substrate material. Utilizing these insights, we were able to manufacture thermoresponsive poly(glycidyl ether) brushes on moderately hydrophobic polystyrene and polycarbonate as well as on rather hydrophilic polyethylene terephthalate and tissue culture-treated polystyrene substrates. We further show that the temperature-dependent switchability of the brush coatings is not only dependent on the cloud point temperature of the block copolymers, but also markedly governed by the hydrophobicity of the surface-bound benzophenone anchor and the subjacent substrate material. Our findings demonstrate that the design of amphiphilic thermoresponsive block copolymers is crucial for their phase transition characteristics in solution and on surfaces.

## 1. Introduction

Coatings based on thermoresponsive polymers consistently prove to be a valuable foundation for cell sheet engineering purposes [1,2,3]. Due to their physical response to changes in temperature under aqueous conditions, such coatings can switch from a rather hydrophobic, protein- and cell-adhesive state into a more hydrophilic, protein- and cell-repellant state upon cooling [4,5]. This phase transition is characterized by an increase in coating hydration, which is usually accompanied by the swelling of the thermoresponsive polymer layer. The temperature at which this transition occurs is therefore often referred to as the volume phase transition temperature (VPTT). In general, the switchability of thermoresponsive coatings depends on a variety of factors, specifically the hydrophilicity of the polymeric coating material, the coating architecture and thickness, the nature of the substrate material and the composition of the aqueous medium. There are numerous reports of thermoresponsive coatings which, under standard cell culture conditions at 37 °C, allow the adhesion and proliferation of mammalian cells to confluent monolayers, or cell sheets, which can subsequently be harvested in a triggered manner by decreasing the temperature below the VPTT of the coating. The cell sheets can then be used as 2D building blocks in tissue engineering and regenerative medicine [6,7]. Besides extensively discussed and investigated poly(*N*-isopropyl acrylamide) (PNIPAm) gel [8] and brush [9] coatings, thermoresponsive brushes based on poly(oxazoline)s (POx) [10,11], poly[oligo(ethylene glycol) methacrylate]s (POEGMAs) [12,13,14] and poly(glycidyl ether)s (PGEs) [15,16] have shown to be among the most suitable thermoresponsive coatings for cell sheet fabrication.

Due to their considerable cell compatibility, coatings based on thermoresponsive PGEs are of great utility in tissue engineering applications [17] and have already proven to be suitable for the culture and temperature-triggered detachment of cell sheets on both gold model surfaces [18,19,20] as well as on applied glass [21] and polystyrene (PS) tissue culture substrates [15,16,22]. Whereas PGEs comprising glycidyl methyl ether (GME) and ethyl glycidyl ether (EGE) as comonomers can be conveniently anchored to gold substrates via a thiol end-group [20,23], the immobilization of PGE brush coatings on applied materials can be achieved via the introduction of surface-specific PGE anchor blocks that either physically and/or covalently bind to the culture substrate. To that effect, PGEs equipped with a positively charged polyamine anchor block were immobilized onto negatively charged glass substrates via electrostatic interactions [21].

To transfer thermoresponsive PGE coatings to most cell culture prevalent, applied tissue culture vessels, we have previously reported the immobilization of PGEs onto rather hydrophobic, untreated PS substrates. In our approach, we utilized block copolymers comprising a hydrophobic BP-based anchor block to facilitate a viable “grafting-to” process for thermoresponsive PGE coatings via physical adsorption from dilute aqueous solution [15,16]. We showed that the directed self-assembly and subsequent covalent UV immobilization of PGE block copolymers via their photo-reactive BP anchor block can be achieved under selective solvent conditions in water below the transition temperature of the block copolymer to yield coatings with a brush-like conformation [15]. Due to the adhesive interaction between PGEs and PS, adsorption from a non-selective solvent, such as ethanol, leads to the formation of ultrathin PGE coatings with a pancake-like conformation [16]. In contrast to non-PGE-adhesive gold and glass substrates, on which PGE brushes undergo a mushroom-to-brush-like transition upon cooling [20,21,23], PGE brushes exhibit a pancake-to-brush-like transition on PS, which can be exploited to harvest a variety of cell sheets comprising different types of human primary cells [15].

Some efforts have been made to develop convenient “grafting-to” procedures to immobilize thermoresponsive coatings onto applied tissue culture substrates via physical adsorption, especially through hydrophobic interactions between copolymers and the culture substrate [24,25,26]. As of yet, thermoresponsive block- and graft-type copolymers comprising substrate specific anchor blocks are among the more well-defined coating materials studied [27,28,29,30,31]. However, studies which correlate the nature of the hydrophobic anchoring domain with its capacity to effectively immobilize thermoresponsive coatings on plastic culture substrates are lacking. The development of versatile anchor blocks is particular worthwhile, especially due to the rather diverse chemical nature and chemical and physical properties of commonly used tissue culture materials. These primarily include untreated PS, tissue culture-treated PS (TCPS), polyethylene terephthalate (PET), polycarbonate (PC), polydimethylsiloxane (PDMS), aliphatic or fluorinated polyolefins (e.g., polyethylene, polypropylene, polyvinylidene fluoride or polytetrafluoroethylene) as well as cellulose-based materials (e.g., cellulose acetate or nitrocellulose). With only few exceptions (e.g., polyether sulfone, polytetrafluoroethylene), most polymeric materials commonly used for biomedical applications, such as tissue engineering, comprise aliphatic groups susceptible to C,H-insertion crosslinking (CHic) reactions with BP and its derivatives [24,32,33,34,35]. It therefore comes as no surprise that BP photo-chemistry has been a proven remedy, especially in the fields of bioconjugation and surface functionalization [36]. Hence, CHic photo-chemistry allows for the convenient utilization of BP as an immobilizing agent suitable for a vast variety of polymeric materials comprising otherwise rather unreactive aliphatic C-H groups [37].

Regarding their practical utility for cell sheet fabrication purposes, culture substrate materials require to be transparent, in order to monitor cell growth, and need to be readily available in different forms and geometries depending on the demands of the tissue to be engineered. Various formats of PS and TCPS culture substrates, such as dishes, well-plates and flasks, constitute the most common standard culture materials. PC and PET are also available as flexible, porous membranes, which can increase the rate of hydration of a thermoresponsive polymer coating and, hence, assist cell sheet fabrication. Additionally, such membranes can also be exploited for the direct transfer of detaching cell sheets onto in vitro tissue culture scaffolds or for the regeneration of tissues in vivo. Whereas both “grafting-from” and “grafting-to” methods have been used to manufacture thermoresponsive PNIPAm coatings on most of the common culture substrate materials, reports on promising POx and POEGMA brush coatings are still mainly limited to glass substrates [10,11,12,13,14,21].

In the present work, we investigate the effect of BP anchor blocks with different linker length and chemistry between the photo-reactive BP moiety and the glycidyl ether-based backbone on the thermoresponsive properties of the resulting PGE-X-BP block copolymers in solution. Based on their distinct temperature-dependent aggregation behaviors we developed a robust, broadly applicable “grafting-to” process for the self-assembly of PGE block copolymers on PS, PC, PET and TCPS culture substrates. Finally, we evaluate the grafting efficiency and the temperature-triggered switchability of the brush coatings with respect to the BP anchor block as well as the substrate material.

## 2. Materials and Methods

### 2.1. Materials

Glycidyl methyl ether (GME, ≥85%), ethyl glycidyl ether (EGE, ≥98%), 4-hydroxybenzophenone (4-HBP, ≥98%) and cysteamine hydrochloride (Cys-HCl, ≥98%) were purchased from TCI GmbH (Eschborn, Germany). Allyl glycidyl ether (AGE, ≥99%), tetraethylammonium bromide (N(Oct)_4_Br, 98%), triisobutylaluminum (Al(*i*-Bu)_3_, 1.0 M in hexanes), 4-benzoylbenzoic acid (4-CBP, 99%), 2,2-dimethoxy-2-phenylacetophenone (DMPA, 99%), *N,N*-dimethylformamide (DMF, 99.8%, anhydrous), benzophenone (BP, 98%), sodium lumps (Na), toluene (99.8%), methanol (99.8%) and ethanol (tech.) were supplied by Merck KGaA/Sigma Aldrich (Darmstadt/Steinheim, Germany). Epichlorohydrin (ECH, 98%) was purchased from abcr GmbH (Karlsruhe, Germany). *N*-(3-dimethylaminopropyl)-*N*′-ethylcarbodiimide hydrochloride (EDC-HCl, ≥99%), sodium hydroxide (NaOH, ≥98%), sodium sulfate (Na_2_SO_4_, 99%), molecular sieve (3 Å) and calcium hydride (CaH_2_, 93%) were supplied by Carl Roth GmbH + Co. KG (Karlsruhe, Germany). Diethyl ether (Et_2_O, 99%) was purchased from VWR Chemicals (Leuven, Belgium). Silicon wafers were supplied by Silchem GmbH (Freiberg, Germany). Toluene was pre-dried via the solvent system MB SPS-800 from M. Braun GmbH (Garching, Germany), refluxed with elemental sodium and a pinch of BP and subsequently distilled on activated molecular sieve directly before use. GME, EGE and AGE were dried over CaH_2_, distilled and stored over activated molecular sieve before use. Et_2_O and ethanol were distilled before use to remove impurities.

### 2.2. Methods

^1^H and ^13^C NMR spectra were recorded on a Joel ECX at 400 or 500 and 100 or 126 MHz, respectively, and processed with the software MestReNova (version 7.1.2). Chemical shifts were reported in δ (ppm) and referenced to the respective deuterated solvent peak (CDCl_3_). GPC was conducted on an Agilent 1100 Series instrument in THF as the eluent at concentrations of 3.5 mg mL^−1^ and a flow rate of 1 mL min^−1^ at 25 °C. Three PLgel mixed-C columns (Agilent, Waldbronn, Germany) with dimensions of 7.5 mm × 300 mm and a particle size of 5 µm were used in-line with a refractive index detector. Calibration was performed with polystyrene (PS) standards from PSS (Mainz, Germany) and calculation was performed with PSS Win-GPC software. ESI-ToF mass spectral data were obtained on an Agilent 6210 ESI-TOF (Agilent Technologies, Santa Clara, CA, USA) spectrometer at flow rates of 4 mL min^−1^ and a spray voltage of 4 kV.

Dynamic light scattering (DLS) was performed on a Zetasizer Nano-ZS analyzer (Malvern Instruments, Malvern, United Kingdom) equipped with a 50-mW frequency doubled DPSS Nd:YAG laser (λ = 532 nm) in Milli-Q water at 10, 20 and 37 °C. Measurements were performed in two consecutive triplicates using Quartz cuvettes supplied by Hellma Analytics GmbH (Müllheim, Germany) and each sample was equilibrated for at least 10 min at each temperature prior to measurements. Turbidimetry measurements were performed on a Lambda 950 UV-Vis spectrometer at λ = 500 nm with a PTP 6 Peltier Temperature Programmer from Perkin Elmer (Waltham, MA, USA). Cloud point temperatures (CPTs) were determined in Milli-Q water employing heating/cooling rates of 0.5 °C min^−1^ with a data point recording every 0.2 °C. The temperature-dependent transmittance of the aqueous polymer solutions was measured for at least four up and down cycles and the CPT was defined as the temperature at the inflection point of the normalized transmittance versus temperature curves.

Spin-coating was performed using a WS-650-23 spin-coater from Laurell Technologies (North Wales, PA, USA). Silicon wafers (11 mm × 11 mm) were coated at 3000 rpm for 60 s using 50 µL of either a 1% (*w*/*w*) solution of PS in toluene, a 1.0% (*w*/*w*) solution of PC in THF or a 0.5% (*w*/*w*) solution of PET in HFIP. TCPS substrates were fabricated by irradiation of PS coated silicon wafers in a Beltron UV chamber (Hg lamp, 850 W) from Beltron GmbH (Rödermark/Urberach, Germany) at a distance of 12 cm (~25 mW cm^−2^) for 30 s. The oxidized samples were extracted in ethanol for 1 d, subsequently washed with Milli-Q water and directly used for coating experiments within 1–2 d. Static water contact angles (CAs) were measured with an OCA contact angle system from DataPhysics Instruments GmbH (Filderstadt, Germany) and fitted with the software package SCA202 (version 3.12.11) using the sessile drop method. CAs were determined before and after surface functionalization at ambient 20 °C and at 37 °C under humid conditions in a measurement chamber. A drop of Milli-Q water (2 μL) was placed onto the respective surface and CAs were determined with the Young-Laplace model. For each substrate, CAs were measured on at least five different spots to test for the homogeneity of the sample and at least six independent substrates (n = 6) to test for reproducibility. The dry layer thickness of the polymer coatings was determined by spectroscopic ellipsometry (SE) at an incident angle of 70° with a SENpro spectroscopic ellipsometer from Sentech Instruments GmbH (Berlin, Germany). The thickness of the SiO_2_ layer before spin coating and the additional thickness of the spin-coated PS, PC, PET and TCPS layers were determined separately using a Cauchy layer for modelling and respective average values of at least five different spots on the surfaces were taken as fixed values for the subsequent modeling of the adsorbed PGE brush layers. The PGE thickness was measured at wavelengths from 370 to 1050 nm and was fitted using a model consisting of the previously measured layers with fixed parameters, a PGE layer with a fixed refractive index of n = 1.45 and air as the surrounding medium. For photo-immobilization, samples with adsorbed PGE layers were irradiated with UV light using a UV-KUB 2 (λ = 365 nm, irradiance = 25 mW cm^−2^) from Kloé (Montpellier, France) for 160 s, which corresponds to a radiant exposure of 4.0 J cm^−2^.

### 2.3. Monomer Synthesis

The photo-reactive BP-based glycidyl ether comonomers 4-[(2,3-epoxypropoxy)ethoxy] benzophenone (EEBP) and 4-(2,3-epoxypropoxy)benzophenone (EBP) (Figure S1) were synthesized in our previous report [16] and according to a procedure by Jabeen et al. [38], respectively. Details are given in the Supplementary Materials.

### 2.4. Block Copolymer Synthesis and Characterization

PGE block copolymers were synthesized via the sequential monomer-activated anionic ring-opening polymerization (MA-AROP) based our previously published procedure [16]. Three kinds of block copolymers with randomly copolymerized thermoresponsive blocks based on GME and EGE and anchor blocks based on EEBP, EBP and allyl glycidyl ether (AGE) were synthesized. Poly(GME-*ran.*-EGE)-*block*-poly(EEBP) and poly(GME-*ran.*-EGE)-*block*-poly(EBP) block copolymers were directly used for characterization and surface functionalization after purification, whereas poly(GME-*ran.*-EGE)-*block*-poly(AGE) block copolymers were first functionalized with BP moieties via thiol-ene chemistry and subsequent amide coupling based on modified procedures by Heinen et al. [21] and Yu et al. [37], respectively. The comonomer composition and molecular weight characteristics of the PGE block copolymers were determined via NMR spectroscopy and gel permeation chromatography (GPC), respectively. The thermoresponsive properties of the block copolymers in water were characterized by UV-Vis turbidimetry and dynamic light scattering (DLS). Details are given in the Supplementary Materials.

### 2.5. Surface Preparation and Characterization

Silicon wafers (~11 mm × 11 mm) were equipped with thin films (~50 nm) of the culture substrate materials PS, PC and PET via spin coating. TCPS substrates were manufactured by UV/ozone treatment of PS-coated silicon wafers. The native SiO_2_ layer on the silicon wafers and the thickness of the substrate material films were determined separately by spectroscopic ellipsometry (SE) prior to coating. PGE coatings were fabricated by incubation of the coated silicon wafer substrates in dilute (0.25 mg mL^−1^) aqueous/ethanolic solutions of the block copolymers for 1 h. The polymer solutions were subsequently discarded and the surfaces briefly (~30 s) immersed in water to remove excess polymer solution. After drying under a stream of N_2_, the substrates were irradiated with UV light (LED, λ = 365 nm) to covalently immobilize the physically adsorbed PGE layers via their photo-reactive BP anchor blocks. The surfaces were then washed with ethanol to extract non-immobilized PGE chains until the thickness of the coatings was constant (~1 d). The dry thickness of the PGE coatings was measured by SE before and after UV irradiation as well as during extraction with ethanol. The temperature-dependent wettability of the thus obtained PGE brush coatings was characterized by static water contact angle (CA) measurements at 37 and 20 °C.

### 2.6. Statistical Evaluation

Data illustration and analysis was performed using the software OriginPro2018. Static water CAs at 37 and 20 °C were statistically compared using the unpaired t-test for two independent sample sets following a normal distribution and assuming different variances (*, *p* < 0.05; **, *p* < 0.01; ***, *p* < 0.005; ****, *p* < 0.001). Normal distribution was assessed using the Shapiro-Wilk test (*p* < 0.05).

## 3. Results

### 3.1. Block Copolymer Synthesis

**Scheme 1:**
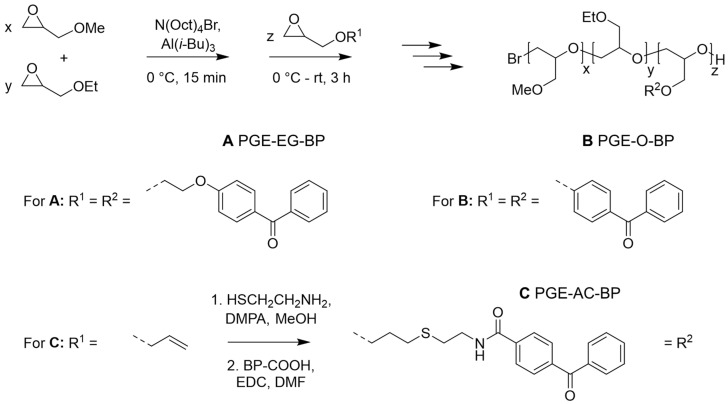
Schematic illustration for the synthesis of thermoresponsive GME/EGE-based PGE block copolymers **A**, **B**, **C** comprising different short, photo-reactive BP-based anchor blocks via the sequential MA-AROP. Different BP units were either introduced directly (copolymers **A** and **B**), or by post-modification of allyl-groups with cysteamine hydrochloride via thio-ene chemistry and further amidation with 4-benzoylbenzoic acid (copolymer **C**).

To study the effect of the chemical composition and hydrophobicity of a BP anchor block on the fabrication of thermoresponsive PGE brush coatings and to extend the applicability of PGE brushes from PS [15,16] to other relevant culture substrate materials, we synthesized PGE block copolymers with different BP anchor blocks (Scheme 1). Therefore, two BP monomers with different linker chemistry between the photo-reactive BP and the polymerizable glycidyl ether moiety or, alternatively, post-modification of a short allyl-precursor block have been applied. According to our previously established synthesis strategy for PGE-X-BP block copolymers, the sequential MA-AROP yields PGE block copolymers with a random [39], high-molecular-weight, thermoresponsive GME/EGE block and a short, rather hydrophobic, photo-reactive BP-based anchor block [16]. In the PGE-X-BP nomenclature X indicates the spacer between the polymer backbone and the BP unit.

We have shown previously that copolymers **A** are accessible via copolymerization applying the photo-reactive comonomer EEBP yielding poly(GME-*ran.*-EGE)-*block*-poly(EEBP) block copolymers, which can be used to fabricate ultrathin sub-nm PGE coatings [16] as well as PGE brush coatings [15] on hydrophobic, untreated PS culture substrates. Well-defined block copolymers with a number average molecular weight (*M*_n_) close to 30 kDa and an average of 3.3 BP repeating units per polymer chain (Table 1, entry 1, **A**1:3) were prepared. Since this block copolymer is equipped with an ethylene glycol (EG) spacer, it is herein referred to as PGE-EG-BP (Scheme 1).

To modify the hydrophilic-hydrophobic asymmetry of the block copolymers, we synthesized PGEs using the more hydrophobic comonomer EBP, which lacks the flexible, hydrophilic EG spacer, to yield the photo-reactive anchor block. As summarized in Table 1 (entries 2 and 3, **B**1:3 and **B**1:1), we obtained well-defined poly(GME-*ran.*-EGE)-*block*-poly(EBP) block copolymers with comparable molecular weights close to 30 kDa and an average of 4–5 BP units per polymer chain which are referred to as PGE-O-BP (Scheme 1). PGE-O-BPs with GME:EGE comonomer ratios of 1:3 (**B**1:3) and 1:1 (**B**1:1) were synthesized to obtain copolymers with phase transition temperatures around room temperature (20 °C) [39] and in the vicinity of 30 °C [40], respectively, both suitable for cell sheet fabrication.

Based on a post-modification approach [21], we synthesized copolymers **C** with GME:EGE comonomer ratios of 1:3 (**C**1:3) and 1:1 (**C**1:1) using AGE as comonomer of the anchor block. The two-step post-functionalization of the allyl groups via thiol-ene “click” chemistry and subsequent amide coupling of a carboxy-functional BP derivative yielded well-defined poly(GME-*ran.*-EGE)-*block*-poly(AC-BP) block copolymers **C** with allyl-cysteamine-based anchor block spacers (Table 1, entries 4 and 5, **C**1:3 and **C**1:1). These block copolymers are herein referred to as PGE-AC-BP (Scheme 1).

Having block copolymers **A** (PGE-EG-BP), **B** (PGE-O-BP) and **C** (PGE-AC-BP) comprising three different kinds of BP anchor blocks at hand, we examined their thermoresponsive properties and aggregation behavior in aqueous solution in order to assess their suitability for self-assembly onto various applied tissue culture substrates.

### 3.2. Thermoresponsive Properties of Block Copolymers

Just like PNIPAm, POx and POEGMA, thermoresponsive PGE copolymers comprising GME and EGE exhibit a lower critical solution temperature (LCST) in water [18,40,41]. Due to this LCST-type phase transition, such PGEs are soluble in aqueous media below a critical temperature, whereas they undergo a coil-to-globule transition to form turbid solutions when the temperature is raised above their so-called cloud point temperature (CPT). Similar to various POx and POEGMA copolymers [42,43], the CPT of thermoresponsive PGEs can be adjusted via the GME:EGE comonomer ratio and the sharp phase transition is fully reversible and exhibits little to no thermal hysteresis [19,39,40,44]. In addition, PGEs exhibit a concentration-dependent as well as a strongly molecular weight-dependent CPTs in the range between 1 and 30 kDa [39,44].

**Figure 1:**
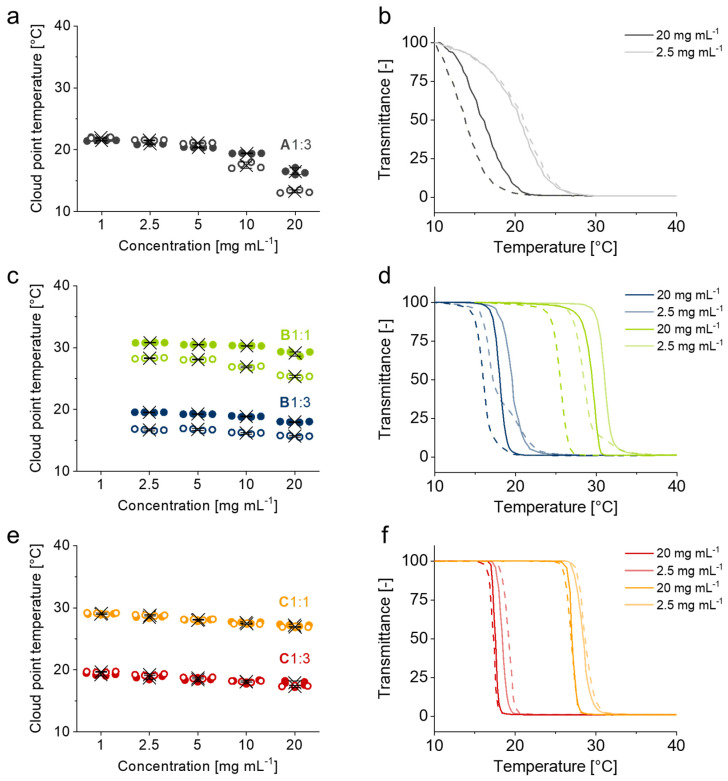
Concentration-dependent CPT (left) from 1 to 20 mg mL^−1^ and representative normalized transmittance curves (right) at 2.5 and 20 mg mL^−1^ of **A**1:3 (**a**,**b**) [15], **B**1:3 and **B**1:1 (**c**,**d**) and **C**1:3 and **C**1:1 (**e**,**f**) in water determined by turbidimetry at 500 nm. Full dots and continuous lines represent heating cycles. Hollow dots and dashed lines represent cooling cycles. CPTs are plotted for each consecutive heating/cooling cycle together with their mean values (cross) and 90% confidence intervals (whiskers) (n = 4).

The presence of largely hydrophobic BP-containing blocks in copolymers **A**, **B** and **C** can impact their general solubility in aqueous solution and the thermoresponsiveness of the PGE block in particular. To investigate the thermoresponsive properties of PGE-X-BP block copolymers in water, we determined their CPTs at concentrations between 1 and 20 mg mL^−1^ by turbidimetry at 500 nm. The concentration-dependent CPTs as well as representative, normalized transmittance curves are illustrated in Figure 1. Overall, CPTs of PGE-X-BPs with a GME:EGE comonomer ratio of 1:3 (**A**1:3, **B**1:3, **C**1:3) are close to room temperature at around 20 °C, whereas PGE-X-BP block copolymers with a comonomer ratio of 1:1 (**B**1:1, **C**1:1) exhibit CPTs around 30 °C. Thus, the CPTs of the block copolymers are well within the targeted range for cell culture applications (20–37 °C) and comparable to the ones of PGE polymers lacking the BP anchor block [39,40]. As reported previously [15] and illustrated in Figure 1a,b, PGE-EG-BP block copolymer **A**1:3, which is equipped with a BP anchor block comprising a flexible and hydrophilic spacer, exhibits a concentration-dependent CPT (ΔCPT_conc._ ~ 5–8 °C), a relatively broad phase transition regime (ΔCPT_reg._ > 10 °C) and thermal hysteresis (ΔCPT_hyst._ ~ 2–3 °C) at high concentrations (10–20 mg mL^−1^). In comparison, PGE-O-BP block copolymers **B**1:3 and **B**1:1 (Figure 1c,d), which are equipped with a BP anchor block comprising a less flexible and more hydrophobic spacer, exhibit a less concentration-dependent CPT (ΔCPT_conc._ ~ 2–3 °C), a narrower phase transition regime (ΔCPT_reg._ ~ 5–10 °C) and a rather concentration-independent thermal hysteresis (ΔCPT_hyst._ ~ 2–4 °C) within the examined concentration range. Surprisingly, the CPTs of PGE-AC-BP block copolymers **C**1:3 and **C**1:1 (Figure 1e,f) show little concentration dependence (ΔCPT_conc._ ~ 1–2 °C), a significantly narrower phase transition regime (ΔCPT_reg._ ~ 2–4 °C) and no considerable thermal hysteresis (ΔCPT_hyst._ ~ 0 °C). Unlike PGE-EG-BP and PGE-O-BP, PGE-AC-BP block copolymers exhibit phase transition characteristics which closely resemble and are virtually indistinguishable from those of poly(GME-*ran.*-EGE) copolymers without any hydrophobic BP-based anchor block [39].

**Figure 2:**
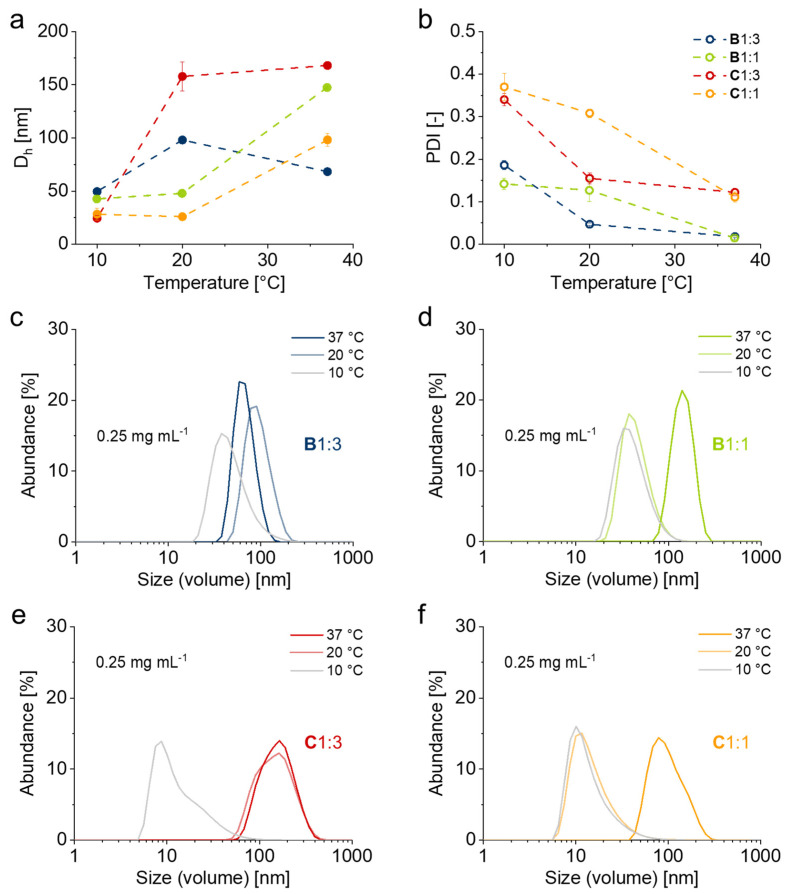
Temperature-dependent volume average hydrodynamic diameter *D*_h_ (**a**), polydispersity PDI (**b**) and representative particle size distributions of **B**1:3 (**c**), **B**1:1 (**d**), **C**1:3 (**e**) and **C**1:1 (**f**) in water at 37, 20 and 10 °C and 0.25 mg mL^−1^ determined by DLS. (Error bars indicate standard deviation (SD); n = 6).

To investigate the coil-to-globule transition of the block copolymers and to further get an insight into their aggregation behavior, we measured their particle sizes in water at 10, 20 and 37 °C using DLS. Average particle sizes and their representative distributions of PGE block copolymers measured under dilute conditions at 0.25 mg mL^−1^ are summarized in Figure 2. As illustrated in Figure 2a–c, the size distribution of **B**1:3 mesoglobules above the polymer’s CPT at 37 °C is rather narrow with an average around 70 nm. Around the CPT of **B**1:3 at 20 °C, the hydrodynamic diameter (*D*_h_) of the aggregates increase to about 100 nm, which indicates a temperature-induced swelling of the mesoglobules. Below the CPT at 10 °C, the particle size decreases to an average of 50 nm. A similar behavior was observed for the temperature-dependent particle sizes of **B**1:1. Whereas the polymer forms mesoglobules with a rather narrow size distribution and average *D*_h_ between 100–200 nm above its CPT at 37 °C, particle sizes decrease to ~40–50 nm below the polymer’s CPT at 20 as well as 10 °C (Figure 2a,b,d). As illustrated in Figure S5a–d, particle sizes of **B**1:3 and **B**1:1 at 2.5 mg mL^−1^ closely resemble the distributions at 0.25 mg mL^−1^ (Figure 2a–d). Furthermore, comparative measurements at 2.5, 0.25 and 0.025 mg mL^−1^ show that both **B**1:3 and **B**1:1 exhibit a distinct aggregation behavior which is almost independent on the polymer concentration within the measured range (Figure S6). As apparent from Figure S6a,b, the polymer concentration merely affects the size of the mesoglobules above the polymers’ CPT at 37 °C. In addition, the increase in *D*_h_ of **B**1:3 at 20 °C with decreasing polymer concentration strongly suggests the swelling of the mesoglobules, which apparently dissociate into smaller-sized aggregates upon cooling below the polymer’s CPT at 10 °C (Figure S6a,c,e,g).

In contrast to **B**1:3 and **B**1:1, block copolymers **C**1:3 and **C**1:1 generally exhibit broader particle size distributions (Figure 2b,e,f). Above and around their CPTs, the *D*_h_ of less well-defined **C**1:3 and **C**1:1 mesoglobules is around 100–200 nm and ~100 nm, respectively. Below their CTPs, average particle sizes of 10–20 nm can be observed for both PGE-AC-BPs. As compared in our previous reports of PGE [16,39], a substantial fraction of the particles below the CPTs of **C**1:3 and **C**1:1 likely constitute non-aggregated block copolymer unimers [15]. Furthermore, and in contrast to **B**1:3 and **B**1:1, the particle size distributions of **C**1:3 and **C**1:1 are more concentration-dependent, as suggested by the larger sized mesoglobules and increased polydispersity indices (PDIs) at a higher polymer concentration of 2.5 mg mL^−1^ (Figure S5). Whereas **C**1:3 and **C**1:1 do not show a distinct anchor block-induced aggregation behavior at concentrations ≤ 2.5 mg mL^−1^, PGE-O-BPs **B**1:3 and **B**1:1 appear to exhibit a rather well-ordered micellar aggregation below their CPTs in the examined concentration range (Figure S6). Notably, and depending on the concentration, PGE-EG-BP block copolymer **A**1:3 exhibits aggregation properties in water intermediate to, but distinctively different from those of **B**1:3 and **C**1:3 under both, more concentrated, and dilute conditions (Figure S7). Hence, **A**1:3 likely exhibits a less well-defined critical aggregation concentration (CAC) in the range between 2.5 and 0.25 mg mL^−1^ below its CPT at 10 °C.

As indicated in Table 1 and described in the Supplementary Materials, the two-step post-modification which yields block copolymers **C**1:3 and **C**1:1 is not quantitative. Whereas the vast majority of amine groups is converted in the second modification step (~0.2–0.4 residual amine groups per PGE chain), the functionalization of allyl groups with cysteamine units is incomplete. Hence, we assume that an average of about 1.1–1.6 AGE units per PGE chain did either not react with cysteamine or undergo intramolecular side reactions within the anchor block. To estimate the effect of remaining or non-selectively converted allyl groups, we investigated the thermoresponsive properties and aggregation behavior of poly(GME-*ran.*-EGE)-*block*-poly(AGE) block copolymers **1a** and **2a** (Supplementary Materials). As illustrated in Figure S8, the CPTs and respective transition curves of **1a** and **2a** are similar to those of **C**1:3 and **C**1:1 (Figure 1e,f), with **2a** showing even less, or rather no concentration-dependence as compared to **C**1:1 (Figure S8a). However, DLS results clearly revealed a pronounced, but rather poorly defined aggregation of **1a** and **2a** at 10 and 20 °C (Figure S8c,d), respectively, which is well below their CPTs. Due to the significantly less pronounced anchor block induced aggregation of **C**1:3 and **C**1:1, our results strongly suggest a solubilizing effect of the amide group introduced into the block copolymers via post-modification.

Previous adsorption experiments of **A**1:3 on PS culture substrates were performed from dilute aqueous solution (0.25 mg mL^−1^) below the CPT of the polymer at 10 °C in order to allow for the directed self-assembly of PGE brushes [15]. Due to the apparent partial aggregation of **A**1:3 as well as micellar aggregation of **B**1:3 and **B**1:1 under such conditions, we now further optimized this “grafting-to” protocol to enable the efficient self-assembly of PGE block copolymers.

### 3.3. Self-Assembly of Block Copolymer Brushes on Tissue Culture Substrates

**Figure 3:**
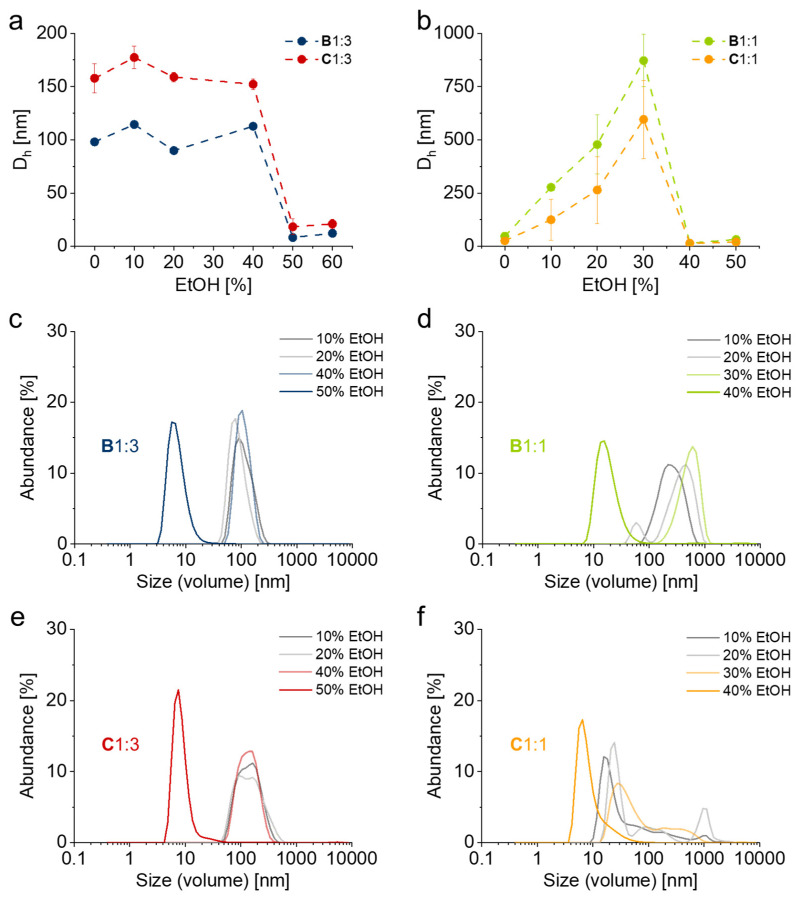
Volume average hydrodynamic diameter *D*_h_ (**a**,**b**) and representative particle size distributions of **B**1:3 (**c**), **B**1:1 (**d**), **C**1:3 (**e**) and **C**1:1 (**f**) in water/ethanol mixtures at 0.25 mg mL^−1^ and ethanol concentrations of 0–50% (*v*/*v*) determined by DLS. (Error bars indicate SD; n = 6).

To facilitate the BP-driven, selective adsorption of PGE block copolymers to various applied culture substrates, we used ethanol as a cosolvent for solubilization of the BP anchor blocks. At the same time, adsorption from dilute aqueous-ethanolic solution should retain its selectivity in order to ensure the hydrophobically-driven, physically stable, BP-directed self-assembly of PGE brush coatings independent on the polymers’ CPT under ambient conditions (~20 °C). Average particle sizes as well as representative size distributions of PGE block copolymers **B**1:3, **B**1:1, **C**1:3 and **C**1:1 in water at 0.25 mg mL^−1^ and at different ethanol cosolvent concentrations are shown in Figure 3. As illustrated, block copolymers **B** and **C** with GME:EGE comonomer ratios of 1:3 exhibit relatively stable particle sizes (100–200 nm) at ethanol concentrations up to 40% (*v*/*v*). However, increasing the amount of ethanol to 50% (*v*/*v*) leads to the dissociation of the large aggregates and the *D*_h_ decreases to ~10 nm, which is well within the range of PGE unimers (Figure 3a,c,e) [16,39]. In addition, visual examination of polymer solutions with ethanol concentrations ranging from 40% to 50% (Figure S9) revealed a recognizable switch in turbidity at ethanol concentrations around 46% for **B**1:3, which further indicates the dissociation of aggregates. As further illustrated in Figure 3, both block copolymers **B** and **C** with GME:EGE comonomer ratios of 1:1 exhibit growing particle sizes with increasing amounts of cosolvent at ethanol concentrations up to 30% (*v*/*v*). Notably, the average *D*_h_ of the aggregates is significantly larger for **B**1:1 (Figure 3b,d), whereas **C**1:1 exhibits almost bimodal distributions with particle sizes around 20–40 nm as well as around 200–1000 nm (Figure 3b,f). For both polymers, increasing the ethanol concentration to 40% leads to the dissociation of aggregates to unimers with a *D*_h_ around 10 nm, which is indicated by a subtle change in turbidity at 32% ethanol for **B**1:1 (Figure S9).

**Figure 4:**
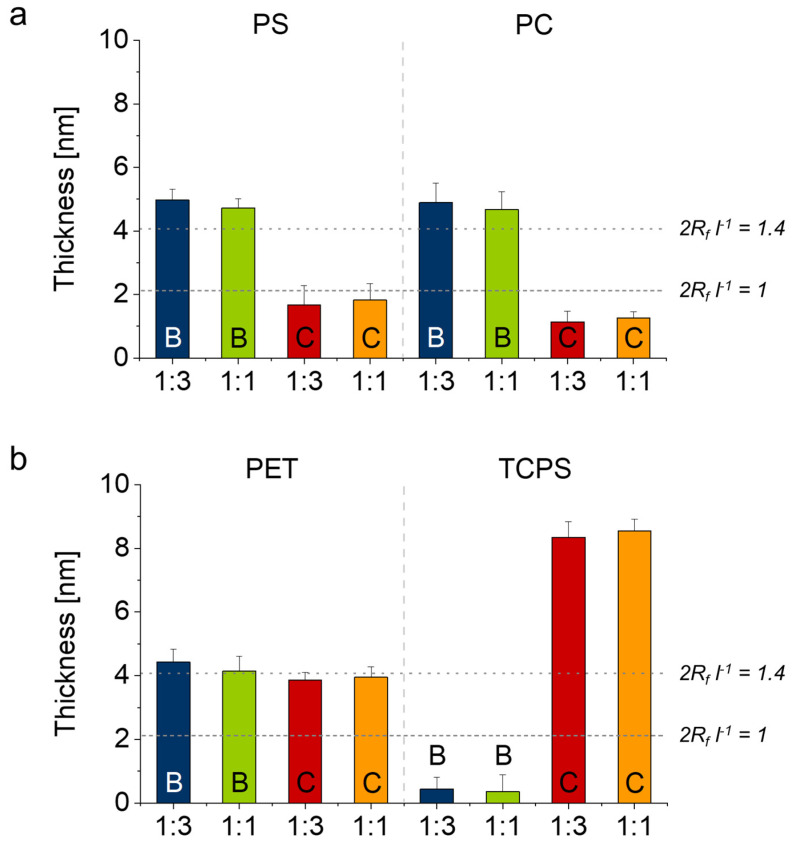
Average dry layer thickness of self-assembled, UV-immobilized PGE coatings **B**1:3 (blue), **B**1:1 (green), **C**1:3 (red) and **C**1:1 (orange) on PS- and PC- (**a**) as well as PET- and TCPS-coated (**b**) silicon wafer model substrates determined by SE. Horizontal dashed and dotted lines represent the start of the chain overlap regime (*2R_f_ l*^−1^ *≥* 1) and the brush regime at which the substrate surface is completely covered by the polymer coating (*2R_f_ l*^−1^ ≥ 1.4) under bad solvent conditions at 37 °C. (Error bars indicate SD; n = 6).

The CACs with respect to the cosolvent ethanol were determined to be 46, 32, 48 and 35% (*v*/*v*) for block copolymers **B**1:3, **B**1:1, **C**1:3 and **C**1:1, respectively. Using dilute solutions (0.25 mg mL^−1^) we performed coating experiments on silicon wafers coated with thin layers (~50–80 nm) of PS, PC, PET and TCPS. These tissue culture model substrates were incubated in PGE solutions for 1 h, briefly washed with water and dried, subsequently irradiated with UV light and washed with ethanol overnight in order to extract non-immobilized PGE chains. The final, dry layer thickness of the obtained PGE coatings measured by SE are illustrated in Figure 4. In summary, block copolymers **B** yield brush coatings with a comparable and uniform dry layer thickness of ~5 nm on PS, PC as well as PET substrates, whereas they did not graft to TCPS substrates. It is worth noting that both **B**1:3 and **B**1:1 adsorbed on all four tissue culture substrates with similar dry layer thickness. Whereas only <1 nm (<20%) is washed off after extraction in ethanol from PS, PET and PC substrates, both block copolymers **B** could be almost entirely washed off from TCPS directly after UV irradiation (Figure 4b), which indicates insufficient covalent immobilization. In contrast, block copolymers **C** only yielded brush coatings on PET and TCPS substrates (Figure 4b). Whereas similar losses in dry thicknesses (<20%) were recorded on these substrates as compared to block copolymers **B**, about 70–80% of the adsorbed coatings were washed off PS and PC substrates after extraction to yield coatings with dry thicknesses <2 nm (Figure 4a).

To estimate the theoretical brush conformation under aqueous conditions, we calculated the degree of chain overlap *2R_f_ l*^−1^ (*R_f_* = Flory radius, *l* = anchor distance) under bad, theta and good solvent conditions (Supplementary Materials) according to our previous reports [15,20,21]. The grafting density and the degree of chain overlap of the PGE brush coatings are summarized in Figures S10 and S11, respectively. As illustrated in Figure S11, the dashed and dotted lines in Figure 4 indicate the start of the chain overlap regime (*2R_f_ l*^−1^ ≥ 1) and the regime at which the substrate surface is completely covered (*2R_f_ l*^−1^ ≥ 1.4), respectively, under bad solvent conditions. This approximates the brush conformation under cell culture conditions above the transition temperature of the PGEs at 37 °C. Consequently, PGE-O-BP block copolymers **B** yield coatings with a brush-like conformation on PS, PC and PET, while PGE-AC-BP block copolymers **C** only form brushes on PET and TCPS tissue culture substrates. Most notably, PGE-AC-BP coatings **C** are significantly thicker, have a markedly higher grafting density and are therefore much further into the extended brush regime (*2R_f_ l*^−1^ ≥ 2.0) than all the other obtained coatings (Figure 4, Figures S10 and S11). Furthermore, all coatings with a brush-like conformation exclusively exhibited higher layer thicknesses than PGE-EG-BP brushes **A** adsorbed from purely aqueous solution as we have reported previously [15]. This demonstrates the effectiveness of our optimized adsorption/immobilization-based “grafting-to” process as well as the selectivity of aqueous/ethanolic solvent mixtures for PGE-X-BP block copolymer self-assembly.

### 3.4. Phase Transition of Poly(glycidyl ether) Brushes

**Figure 5:**
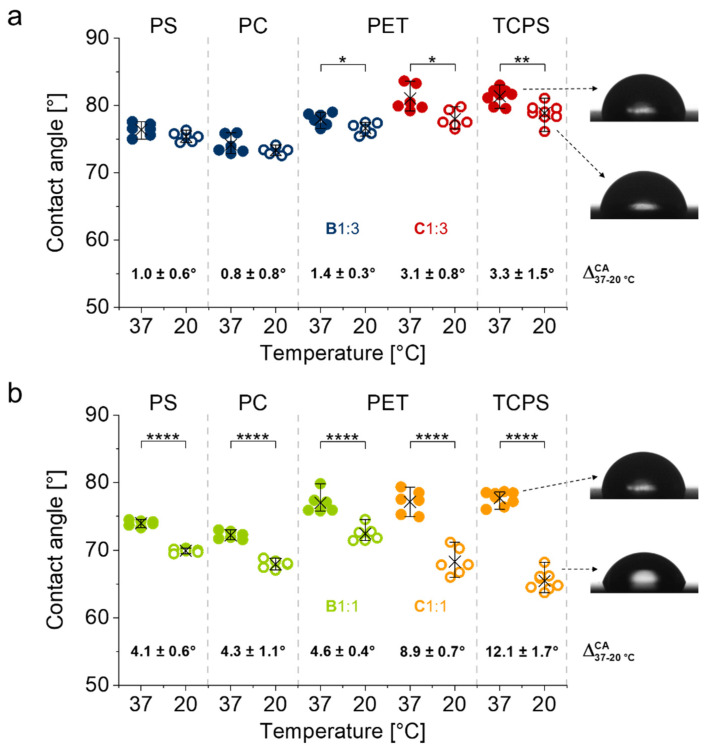
Temperature-dependent wettability of PGE brush coatings **B**1:3 and **C**1:3 (**a**) and **B**1:1 and **C**1:1 (**b**) on PS, PC, PET and TCPS substrates determined at 37 and 20 °C via static water CA measurements. Representative images are shown for C1:3 and C1:1 brushes on TCPS. CAs are plotted for each replicate together with their mean values (cross) and 90% confidence intervals (whiskers) (n = 6). (*, *p* < 0.05; **, *p* < 0.01; ****, *p* < 0.001).

To investigate the switchability of the brush coatings and to assess their potential utility for cell sheet fabrication, we examined their temperature-dependent wettability at 37 °C, which resembles standard cell culture conditions, and under ambient conditions at 20 °C, which corresponds to the targeted cell sheet detachment temperature. Static water CAs of brushes **B**1:3 and **C**1:3 as well as **B**1:1 and **C**1:1 are illustrated in Figure 5a,b, respectively. As illustrated in Figure 5a, **B**1:3 coatings do not show significant wettability differences between 37 and 20 °C on rather hydrophobic PS and PC. However, CAs of **B**1:3 brushes on more hydrophilic PET are slightly, but consistently temperature-dependent. This switchability was also observed and more pronounced for **C**1:3 brushes on both more hydrophilic PET and TCPS substrates. Notably, CAs are generally higher on more hydrophilic PET and TCPS substrates, especially for **C**1:3 brushes, which bear the more hydrophilic BP anchor block.

Whereas the thermo-switchability of brushes with a GME/EGE comonomer ratio of 1:3 and CPTs around 20 °C is rather subtle, a significant temperature-dependent difference in wettability was observed for PGE brushes with a GME/EGE comonomer ratio of 1:1 and CPTs around 30 °C. As illustrated in Figure 5b, the switchability of **B**1:1 is comparable on PS, PC and PET substrates with average ΔCAs of ~4–5 °C between 37 and 20 °C. This switchability is significantly more pronounced for **C**1:1 on PET and, especially, TCPS. Notably, CAs of PGE brushes are generally higher at 37 °C on the more hydrophilic PET and TCPS substrates than on more hydrophobic PS and PC substrates. This is also true for CAs of **B**1:1 on PET at 20 °C. In contrast, CAs of **C**1:1 brushes are markedly lower and decrease with increasing substrate hydrophilicity at 20 °C. Whereas the switchability of **C**1:3 and **C**1:1 on TCPS substrates could be attributed to the significantly higher grafting densities and thicknesses of these coatings, the temperature-dependent changes in wettability on PS, PC and PET substrates, on which all of the investigated coatings have comparable grafting densities and thicknesses, indicate that the switchability of PGE brushes is influenced by the chemistry and hydrophobicity of the BP-based anchor block as well as the subjacent substrate material.

## 4. Discussion

Thermoresponsive PGE-X-BP block copolymers comprising the comonomers GME and EGE and bearing short, photo-reactive BP-based anchor blocks can be synthesized via the MA-AROP. The sequential copolymerization of the photo-reactive BP monomer EEBP, which is equipped with an EG spacer between the glycidyl ether and the BP moiety, has already been presented in one of our previous reports and yields PGE-EG-BP block copolymers [16]. Copolymerization of AGE anchor units and their subsequent post-functionalization via thiol-ene “click” chemistry has also been proven as a facile route to thermoresponsive PGE block copolymers [21]. In the present study, we modified AGE-bearing PGEs via thiol-ene chemistry and subsequent amide coupling to obtain photo-reactive PGE-AC-BP block copolymers with an allyl-cysteamine-based BP anchor block spacer. We further extended the range of copolymerizable BP monomers to the photo-reactive comonomer EBP, which, unlike EEBP, is not equipped with an EG spacer and allows for the synthesis of PGE-O-BP block copolymers with a hydrophobic, less flexible BP anchor block.

**Scheme 2:**
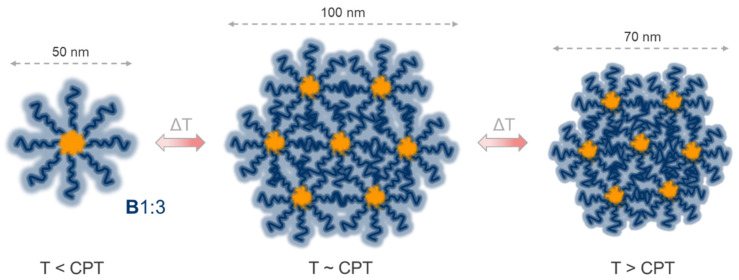
Schematic illustration of the temperature-dependent aggregation behavior of PGE-O-BP block copolymer **B**1:3 in water under dilute conditions at 0.25 mg mL^−1^.

Similar to PGEs without hydrophobic anchor blocks [39,40], PGE block copolymers with GME:EGE comonomer ratios of 1:3 (**A**1:3, **B**1:3, **C**1:3) and 1:1 (**B**1:1, **C**1:1) exhibit CPTs in the realm of 20 and 30 °C, respectively (Figure 1). PGE-O-BPs equipped with the least flexible and most hydrophobic BP anchor block, exhibit CPTs with only little concentration dependence, but consistent thermal hysteresis as well as slightly broader phase transition regimes than PGEs without a BP anchor block, which is especially pronounced during cooling cycles (Figure 1c,d). Both, thermal hysteresis and broader phase transition regimes are likely caused by the distinct aggregation behavior of PGE-O-BPs in aqueous solution, which slows down the dissociation of the mesoglobules formed above the CPTs. In addition, particle size distributions revealed that PGE-O-BPs form well-defined aggregates at 10 °C, which is well below their respective CPTs (Figure 2a–d). This suggests an association of the block copolymers into micellar aggregates comprising hydrophobic BP cores. The distinct increase in *D*_h_ of **B**1:3 from 37 to 20 °C further indicates that PGE mesoglobules formed at 37 °C undergo swelling around the CPT (~20 °C) before dissociating into smaller micellar aggregates at 10 °C (Figure 2a,c), which becomes more pronounced at lower concentration (Figure S6). These results suggest that the hydrophobic BP anchor blocks of both **B**1:3 and **B**1:1 stabilize multimolecular micellar aggregates of both block copolymers below their respective CPTs. Furthermore, the well-defined mesoglobules formed above the CPTs of the PGE block copolymers (Figure 2a–d, Figures S5 and S6) most likely consist of multi-micellar aggregates stabilized by the hydrophobic interactions between the thermoresponsive PGE blocks. Based on the data shown in Figure 2 and Table 2, the temperature-dependent aggregation behavior of block copolymer **B**1:3 is schematically illustrated in Scheme 2.

As visualized in Scheme 2, the apparent molecular weight of PGE-O-BP micelles is significantly higher than the molecular weights of **B**1:3 and **B**1:1. Whereas this likely enhances intermolecular aggregation during heating, the dissociation of the formed mesoglobules during cooling is presumably decelerated by the more compact structure of the “star-like” micellar particles. This is likely the cause for the pronounced thermal hysteresis of **B**1:3 and **B**1:1 (Figure 1). In recent literature, thermoresponsive copolymers comprising “short”, permanently hydrophobic blocks and their temperature-dependent aggregation behavior in water have been subject of several studies. Among others, copolymers equipped with aromatic blocks, such as PS, are especially suitable for comparison with the BP-based block copolymers investigated in the present study. For example, below their CPTs, PNIPAm copolymers with 2–5 kDa terminal PS blocks have been reported to exhibit CMCs in the range of ~1–30 µg mL^−1^, depending on the molecular weights of both PNIPAm and PS blocks [45,46,47]. It was further shown, that thermoresponsive poly[(methoxy diethylene glycol) methacrylate] and POEGMA both equipped with terminal PS blocks of ~1–1.5 kDa exhibit CMCs in water at ~430 and ~50–200 µg mL^−1^, respectively [48,49,50]. Ge et al. investigated the aggregation behavior of PNIPAm (~25–35 kDa) equipped with terminal dendritic G_2_ (0.7 kDa) and G_3_ (1.6 kDa) poly(benzyl ether) (PBE) blocks [51], which are similar to the herein reported EBP-based anchor blocks in both chemistry and molecular weight (Table 2). They showed that the dendritic-linear block copolymers exhibit CMCs ≤ 10 µg mL^−1^ [51]. Overall, the aggregation behavior of PGE-O-BP block copolymers is in line with literature reports. The average molecular weights, temperature-dependent *D*_h_ at 0.25 mg mL^−1^ as well as the estimated CMCs of all the block copolymers investigated in our study are summarized in Table 2.

The CPTs and phase transition behavior of PGE-AC-BPs are virtually indistinguishable from PGEs without an additional BP anchor block [16,39]. CPTs of both **C**1:3 and **C**1:1 show little concentration-dependence, exhibit sharp phase transition regimes and no discernable thermal hysteresis between heating and cooling cycles (Figure 1). Although the block copolymers contain similar amounts of BP units (Table 1) and the molecular weight of their anchor blocks are markedly higher (Table 2), the solubility of the anchor blocks is vastly different from block copolymers **B**, which can be attributed to the allyl-cysteamine-based spacer between the glycidyl ether backbone and the BP units in the anchor blocks. In fact, DLS particle size measurements confirmed that BP-induced aggregation of the block copolymers plays a minor role and both PGE-AC-BPs exhibit mostly unimeric solutions below their respective CPTs (Figure 2, Figure S5). The increased solubility of the block copolymers in water likely results from the spatial separation of the PGE backbone and BP anchor moieties by the allyl-cysteamine-based spacer, which allows for a more efficient hydration of the glycidyl ether repeating units in the anchor blocks. Further, and likely predominant, the hydration of the anchor blocks is greatly enhanced by the amide bond between the spacer and the BP moiety. As a result, PGE-AC-BPs do not form micellar aggregates and therefore exhibit no CMC in the investigated concentration range (Table 2). As apparent from the phase transition behavior of PGE-EG-BP **A**1:3, which comprises a BP anchor block with a short EG spacer, to a certain extent, this block copolymer combines the characteristics of both **B**1:3 and **C**1:3 (Figure 1, Figure S7). Whereas CPTs show no thermal hysteresis and BP-induced aggregation is a minor factor at lower concentrations (≤5 mg mL^−1^) and temperatures below the CPT, the block copolymer exhibits more concentration-dependent CPTs as well as thermal hysteresis at higher polymer concentrations (≥10 mg mL^−1^). As compared to **B**1:3 and **C**1:3, the moderate length and hydrophobicity of the EG spacer results in a concentration-dependent phase transition behavior as well as a broad phase transition regime. These results therefore clearly show, that the phase transition and aggregation behavior of PGE block copolymers is markedly influenced by the chemical composition and architecture of the rather short BP-based anchor blocks.

To graft PGE brushes to tissue culture substrates from dilute solution (0.25 mg mL^−1^), it is necessary to solubilize the hydrophobic PGE anchor blocks in order to facilitate the efficient adsorption of BP anchor units to the substrate surface. To further afford a viable “grafting-to” process at ambient temperatures, we determined the CACs of PGE-O-BP and PGE-AC-BP block copolymers in water/ethanol mixtures with respect to the cosolvent ethanol (Table 2). Under optimal conditions, PGE block copolymers are mostly present as unimeric solutions and micellar- as well as mesoglobular aggregates are largely dissociated (Figure 3, Figure S9). Coincidentally, the CAC marks the cosolvent concentration at which the hydrophilicity of the solvent mixture still affords the hydrophobically-driven adsorption of BP units to the substrate. The solvent selectivity is hence maximized under these conditions. “Grafting-to” experiments on PS, PC, PET and TCPS tissue culture substrates revealed that the attainable brush grafting density depends on the affinity of the BP-based anchor blocks to the respective substrate material. Most notably, covalently anchored, thermoresponsive PGE brushes could neither be obtained by grafting PGE-O-BPs to hydrophilic TCPS nor PGE-AC-BPs to rather hydrophobic PS and PC substrates (Figure 4, Figure S10). In contrast, PGE-O-BPs were efficiently immobilized on PS and PC to form PGE coatings with a brush-like conformation. Inversely, grafting PGE-AC-BPs to TCPS yielded PGE brushes with the highest grafting densities and dry thicknesses in the range of ~8 nm (Figure 4, Figure S10). Consequently, whereas the rather hydrophobic BP anchor blocks of PGE-O-BPs selectively adsorb to rather hydrophobic PS and PC but show no considerable affinity towards more hydrophilic TCPS, PGE-AC-BPs equipped with a more hydrophilic BP anchor block do not efficiently graft to PS and PC but adsorb to TCPS substrates in a highly selective manner. Surprisingly, both types of block copolymers yield PGE brushes with thicknesses and grafting densities comparable to PGE-O-BPs on PS and PC on rather hydrophilic PET substrates (Figure 4, Figure S10). The selective adsorption of both kinds of BP anchor blocks on PET suggests that PGE brush self-assembly is not only influenced by the hydrophilicity of the anchor block and the substrate material, but largely dependent on their respective chemical compositions. Whereas all investigated substrates comprise aliphatic as well as aromatic groups which enable the immobilization of photo-reactive PGEs via CHic as well as promote the adsorption of BP-based anchor blocks via hydrophobic and π-π interactions, respectively, non-treated, hydrophobic PS is the only investigated substrate which does not contain additional functional groups comprising heteroatoms. The physical adsorption of the purely ether-based BP anchor blocks of PGE-O-BPs on PS, PC and PET is predominantly driven by hydrophobic and especially π-π interactions. These adhesive interactions are presumably overruled by more repulsive interactions between the BP anchor block and the hydrophilic carbonyl-, carboxyl- and hydroxyl groups on the tissue culture-treated, oxidized TCPS substrate surfaces [52]. Although PC and PET also contain more polar carbonate- and ester groups, the efficient grafting of PGE-O-BPs is not impeded. Besides comprising an allyl-cysteamine-based spacer, PGE-AC-BPs are chemically most distinguished from PGE-O-BPs by the presence of a polar amide group at the BP moieties of the anchor block. Since this amide group is presumably rather well hydrated under the applied grafting conditions, it largely prevents the efficient adsorption on rather hydrophobic PS and PC substrates. In addition, the increased flexibility of the anchor blocks in PGE-AC-BPs could potentially cause an insufficient orientation of BP units towards the rather hydrophobic PS and PC surfaces, which might further contribute to the inefficient immobilization of **C**1:3 and **C**1:1 to these substrates. In stark contrast, the BP amide group can act as an H-bond acceptor as well as donor to carbonyl-, carboxyl- and hydroxyl groups present on a TCPS substrate surface. The self-assembly of PGE-AC-BPs is therefore likely further entropically-driven through the release of water molecules hydrating both the BP amide and the TCPS surface, making the adsorption process highly selective. In contrast to PS and PC, the more hydrophilic PET allows for the selective adsorption of the BP amide anchor, which affords efficient grafting of PGE-AC-BPs.

**Scheme 3:**
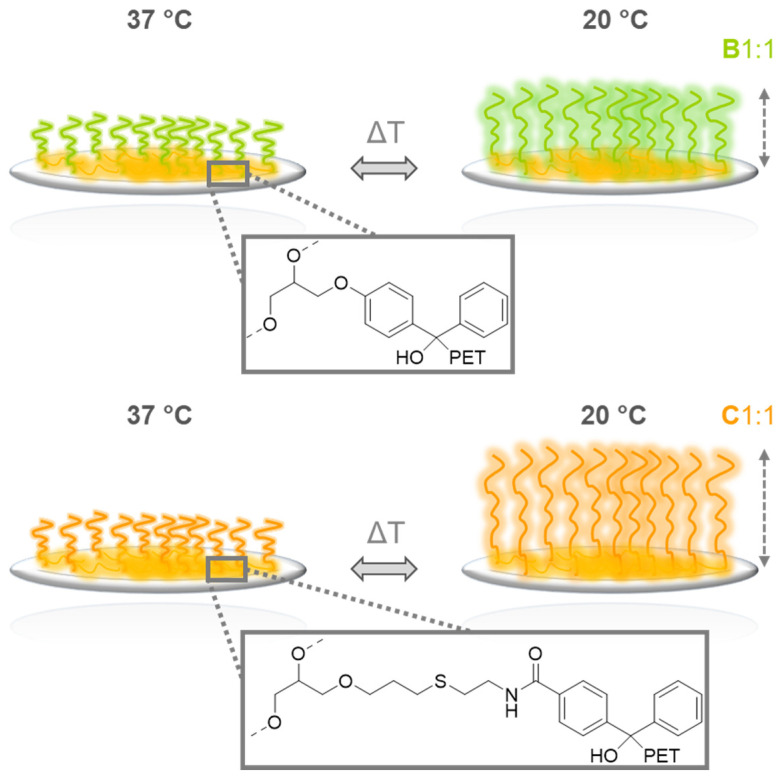
Schematic illustration of the suggested temperature- and anchor-dependent switchability of PGE-O-BP (**B**1:1) and PGE-AC-BP (**C**1:1) brush coatings on PET culture substrates. Compared to **B**1:1, the hydration of **C**1:1 brushes is markedly enhanced as indicated by lower CAs at 20 °C and, correspondingly, more pronounced differences in wettability between 37 and 20 °C as shown in Figure 5b.

Although fundamental studies on gold model surfaces [18,19,20] as well as on applied glass [21] and PS substrates [15,16] have shown that self-assembled PGE monolayers with GME:EGE ratios of 1:3 promote the detachment of mammalian and human cell sheets, so far, the switchability of such coatings could not be detected by means of temperature-dependent changes in their wettability. As illustrated in Figure 5a, PGE brushes based on block copolymers **B**1:3 and **C**1:3 with a CPT around 20 °C do not exhibit temperature-dependent changes in water CAs on hydrophobic PS and PC substrates between 37 and 20 °C. However, slight but significant changes in wettability can be detected on more hydrophilic PET and TCPS substrates. Notably, **C**1:3 brushes tend to exhibit a slightly more distinct switchability than **B**1:3 brushes with similar dry layer thickness on PET substrates. These results indicate that the hydrophilicity of the substrate material as well as the hydrophilicity of the BP-based anchor block influence the phase transition of PGE brushes. This trend is even more pronounced for PGE brushes **B**1:1 and **C**1:1 with CPTs around 30 °C (Figure 5b). Whereas PGE brushes based on **B**1:1 exhibit similar temperature-dependent switchability on PS, PC and PET substrates (ΔCA ~ 4–5 °C), the temperature-dependent differences in water CAs of **C**1:1 are markedly larger on more hydrophilic PET (ΔCA ~ 9 °C) and TCPS (ΔCA ~ 12 °C) substrates. Although the increased switchability of **C**1:1 on TCPS might be attributed to the higher thickness and grafting density as compared to brushes on PS, PC and PET substrates, the results obtained on PET substrates clearly indicate the influence of the BP anchor units on the switchability of PGE brushes. Given that water CAs characterize the hydratability of a coating, temperature-dependent changes in wettability are a measure for the absolute difference in hydration of a thermoresponsive coating. It has been demonstrated before that the hydrophilicity of the substrate material has a significant influence on the properties of thermoresponsive PNIPAm coatings as well as on their performance in cell sheet fabrication [8]. In this context, our results illustrate that hydrophobic culture substrates impede the overall hydratability of thermoresponsive PGE brushes. Since the BP-based anchor units of the block copolymers constitute the interfacial layer between the culture substrate and the thermoresponsive brushes, the chemical composition, linker flexibility and hydrophilicity also play a significant role in overall PGE brush hydration, especially at 20 °C. Temperature-dependent CAs of PGE-O-BP- and PGE-AC-BP-based brushes with comparable dry layer thickness on PET clearly indicate the enhanced switchability of PGE brushes with a longer, more flexible, amide-group containing BP-anchor block spacer. This is most likely due to the elevated hydratability of the coatings mediated by the, overall, more hydrophilic anchor of PGE-AC-BP-based brushes. Scheme 3 schematically illustrates the difference in switchability of **B**1:1 and **C**1:1 on PET culture substrates. As suggested by the temperature-dependent ΔCA (37–20 °C) values of ~5° and ~9° (Figure 5b) for the respective **B**1:1 and **C**1:1 brushes with comparable thickness on PET (Figure 4b), the hydration of **C**1:1 brushes is markedly enhanced at 20 °C as compared to **B**1:1 brushes (Scheme 3).

As shown in Figure 5, both types of PGE brushes exhibit similar average CAs at 37 °C, which indicates similar degrees of hydration above their VPTT (Scheme 3). Whereas **B**1:1 undergoes a moderate swelling upon cooling to 20 °C, **C**1:3 hydrates more efficiently and thus exhibit lower CAs at 20 °C and an enhanced VPT (Figure 5b). This is solely grounded in the distinctly different structure and hydrophilicity of the BP anchor block (Scheme 3).

## 5. Conclusions

Thermoresponsive coatings based on PGE block copolymers can be fabricated through a viable adsorption/immobilization “grafting-to” process. The selectivity of brush self-assembly is markedly governed by the design of the short, photo-reactive BP anchor block and the properties of the culture substrate material. In accordance with their distinct phase transition behavior in water, PGE block copolymers equipped with more, or less hydrophilic anchor blocks graft to various culture substrates with divergent efficiency, which is largely dependent on the chemical nature and hydrophilicity of the substrate material. Utilizing temperature-dependent water CA measurements, we could show that changes in brush wettability are not only determined by the CPT of the PGE block copolymer, but that the switchability of the coatings is also markedly dependent on the hydrophilicity of the substrate material and, most notably, the structure and hydrophilicity of the BP-based anchor block. We believe that our findings will benefit the rational design and translation of thermoresponsive coatings regarding their biomedical application, especially in branches of tissue engineering, such as the fabrication of cell sheets. In addition, the presented coating method constitutes a highly resource-efficient process, especially due to the low amount of required polymer material, the energy-efficiency of the UV immobilization and the scalability of the adsorption process. Furthermore, the process can potentially be transferred to any substrate geometry, which opens up the possibility for the culture and harvest of cell sheets with tailored geometries. Investigations of the phase transition mechanism of the herein developed PGE brush coatings and its implications on cell sheet fabrication are currently under way and will be reported in due time.

## Figures and Tables

**Table 1:** Theoretical (theor.) and experimental (exp.) molecular weight characteristics and comonomer compositions of PGE block copolymers **A**, **B**, **C** synthesized via the sequential MA-AROP.

PGE	*M*_n, theor._ [kDa]	*M*_n, exp._^1^ [kDa]	PDI_exp._^1^	GME:EGE (theor.)	GME:EGE ^2^ (exp.)	BP Units (theor.)	BP Units ^2^ (exp.)
**A**1:3 ^3^	30	26.8	1.05	1:3	1.0:2.8	5.0	3.3
**B**1:3	30	27.1	1.20	1:3	1.0:2.8	5.0	4.8
**B**1:1	30	28.4	1.21	1:1	1.1:1.0	5.0	4.1
**C**1:3	30	28.1	1.18	1:3	1.0:3.1	7.0	5.3
**C**1:1	30	29.8	1.20	1:1	1.0:1.0	7.0	5.1

^1^ Determined by GPC using polystyrene (PS) standards and tetrahydrofuran (THF) as eluent; ^2^ Determined by ^1^H NMR (500 MHz) spectroscopy in deuterated chloroform (CDCl_3_) as solvent; ^3^ Data adapted from [15].

**Table 2:** Average molecular weights *M*_n_ of PGE (GME/EGE) and BP copolymer blocks, average temperature-dependent hydrodynamic diameter *D*_h_ at 10, 20 and 37 °C, CAC with respect to the cosolvent ethanol and estimated CMC of PGE block copolymers.

PGE	*M*_n, PGE_^1^ [kDa]	*M*_n, BP_^1^ [kDa]	*D*_h, 10 °C_^2^ [nm]	*D*_h, 20 °C_^2^ [nm]	*D*_h, 37 °C_^2^ [nm]	CAC [vol-%]	CMC [µg mL^−1^]
**A**1:3	25.8	1.0	67.2 ± 9.9	68.2 ± 16.5	101.2 ± 28.1	-	~250 ^3^
**B**1:3	25.9	1.2	49.6 ± 2.5	98.0 ± 0.8	68.2 ± 0.2	46	<25 ^4^
**B**1:1	27.4	1.0	42.3 ± 1.3	47.5 ± 2.1	147.1 ± 0.9	32	<25 ^4^
**C**1:3	26.0	2.1	24.5 ± 2.6	157.8 ± 13.8	168.1 ± 2.7	48	-
**C**1:1	27.6	2.0	28.3 ± 5.7	26.0 ± 3.2	98.2 ± 5.9	35	-

^1^ Estimated from GPC and ^1^H NMR results; ^2^ Determined by DLS at polymer concentrations of 0.25 mg mL^−1^; ^3^ Corresponds to a CAC rather than a CMC due to the less defined/ordered aggregation behavior of **A**1:3 as compared to **B**1:3 and **B**1:1; ^4^ Estimated from DLS results at concentrations of 2.5, 0.25, 0.025 mg mL^−1^.

## Supplementary Materials

The following are available online at https://www.mdpi.com/2073-4360/12/9/1899/s1, Figure S1: Synthesis of the photo-reactive comonomer EBP, Figure S2: ^1^H-NMR spectrum of the photo-reactive glycidyl ether comonomer EBP, Figure S3: Representative ^1^H-NMR spectrum of PGE-O-BP block copolymer **B**1:1, Figure S4: Representative ^1^H-NMR spectrum of PGE-AC-BP block copolymer C1:1, Figure S5: Temperature-dependent volume average hydrodynamic diameter *D*_h_ (a), polydispersity PDI (b) and representative particle size distributions of **B**1:3 (c), **B**1:1 (d), **C**1:3 (e) and **C**1:1 (f) in water at 37, 20 and 10 °C and 2.5 mg mL^−1^ determined by DLS, Figure S6: Temperature-dependent volume average hydrodynamic diameter *D*_h_ (a, b) and representative particle size distributions of **B**1:3 (c, e, g) and **B**1:1 (d, f, h) in water at 37, 20 and 10 °C and 2.5, 0.25 and 0.025 mg mL^−1^ determined by DLS, Figure S7: Temperature-dependent volume average hydrodynamic diameter *D*_h_ (a, b) and representative particle size distributions of **A**1:3 (c, d), **B**1:3 (e, f) and **C**1:3 (g, h) in water at 37, 20 and 10 °C and 2.5 and 0.25 mg mL^−1^ determined by DLS, Figure S8: Concentration-dependent CPT (a) from 1 to 20 mg mL−1 and representative normalized transmittance curves (b) at 2.5 and 20 mg mL−1 of 1a (red) and 2a (orange) in water determined by turbidimetry at 500 nm and representative temperature-dependent volume average particle size distributions of **1a** (c) and **2a** (d) in water at 37, 20 and 10 °C and 2.5 mg mL-1 determined by DLS, Figure S9: Visual change in turbidity of B1:3 and B1:1 in water/ethanol mixtures at ethanol concentrations of 40–50 and 30–40% (*v*/*v*), respectively, and at a polymer concentration of 0.25 mg mL^−1^, Figure S10: Grafting density of PGE brush coatings **B**1:3, **B**1:1, **C**1:3 and **C**1:1 obtained on PS, PC, PET and TCPS via the adsorption/immobilization “grafting-to” process from aqueous/ethanolic solution at a polymer concentration of 0.25 mg mL^−1^, Figure S11: Theoretically estimated, temperature-dependent brush conformation of **B**1:3, **B**1:1, **C**1:3 and **C**1:1 coatings in water at 37 and 20 °C by means of the degree of chain overlap *2R_f_ l^−1^*.
