# Supplementary material for: Thermoresponsive Poly(glycidyl ether) Brush Coatings on Various Tissue Culture Substrates—How Block Copolymer Design and Substrate Material Govern Self-Assembly and Phase Transition
Source: Polymers (Basel). 2020 Aug 24;12(9):1899. doi: 10.3390/polym12091899 (PMC7563243; doi:10.3390/polym12091899)
Supplement: Supplementary file 1 [file polymers-12-01899-s001.pdf]

Supplementary Materials for the following Article published in the Journal *Polymers* (MDPI)

Thermoresponsive Poly(glycidyl ether) Brush Coatings on Various Tissue Culture Substrates – How Block Copolymer Design and Substrate Material govern Self-assembly and Phase Transition

Daniel David Stöbener [1,2] and Marie Weinhart [1,2,*]

[1] Leibniz Universität Hannover, Institute of Physical Chemistry and Electrochemistry, Callinstr. 3A, 30167 Hannover, Germany; marie.weinhart@pci.uni-hannover.de

[2] Freie Universität Berlin, Institute of Chemistry and Biochemistry, Takustr. 3, 14195 Berlin, Germany; daniel.stoebener@fu-berlin.de

* Correspondence: marie.weinhart@pci.uni-hannover.de

Received: date; Accepted: date; Published: date

1. Monomer Synthesis

The photo-reactive BP monomer 4-(2,3-epoxypropoxy)benzophenone (EBP) was synthesized via base-catalyzed glycidylation of 4-HBP according to a procedure by Jabeen *et al.* [1]. In brief, NaOH (2 g, 50 mmol, 1.7 eq) was added to a solution of 4-HBP (6 g, 30 mmol, 1 eq) in ECH (50 mL, 663 mmol, 22 eq) and the mixture was refluxed for 5 h and stirred at rt overnight (Figure S1). Excess ECH was removed under reduced pressure, the product was dissolved in DCM and washed with water to extract the formed NaCl salt as well as the residual NaOH. The organic phase was dried over Na_2SO_4, filtered, and the crude product was obtained after removing DCM under reduced pressure. The monomer was obtained with a yield of 85% after purification by silica column chromatography in DCM as eluent and stored as a stock solution (~ 50 mg mL^{-1} in DCM) until use. Prior to copolymerization, the solvent of a specified volume of stock solution containing the required amount of EBP was removed by distillation at low pressure in a flame dried 50 mL Schlenk flask. The monomer was subsequently dried under high vacuum and inert conditions and dissolved in dry toluene (~ 100 mg mL^{-1}). The EBP solution was then directly transferred to the sequential monomer-activated anionic ring-opening polymerization (MA-AROP).

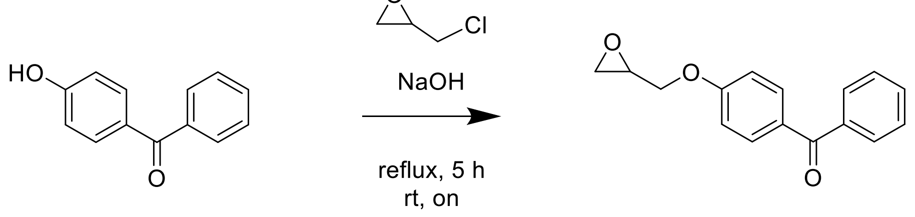

Figure S1. Synthesis of the photo-reactive comonomer EBP.

^{1}H NMR (400 MHz, CDCl$_3$): δ(ppm) = 7.86-7.68 (m, 4H, BP); 7.60-7.38 (m, 3H, BP); 7.04-6.88 (m, 2H, BP); 4.32 (ddd, *J* = 11.0, 3.0, 0.7 Hz, 1H, BPOC$\underline{H_2}$CHOCH$_2$); 4.00 (ddd, *J* = 11.1, 5.8, 0.9 Hz, 1H, BPOC$\underline{H_2}$CHOCH$_2$), 3.37 (ddt, *J* = 5.8, 4.3, 2.8 Hz, 1H, BPOCH$_2$C$\underline{H}$OCH$_2$), 2.92 (ddd, *J* = 5.0, 4.1, 0.9 Hz, 1H, BPOCH$_2$CHOC$\underline{H_2}$), 2.77 (ddd, *J* = 4.9, 2.7, 0.8 Hz, 1H, BPOCH$_2$CHOC$\underline{H_2}$); ^{13}C NMR [^{1}H] (101 MHz, CDCl$_3$): δ(ppm) = 195.6 ($\underline{C}$=O); 162.1 ($\underline{C}$OCH$_2$CHOCH$_2$); 138.2, 132.6, 132.1, 130.7, 129.8, 128.3, 114.2 (BP); 69.0 (-O$\underline{C}$H$_2$CHOCH$_2$); 50.0 (-OCH$_2\underline{C}$HOCH$_2$); 44.7 (-OCH$_2$CHO$\underline{C}$H$_2$); ESI-ToF: m/z = 255.1 [M + H]$^+$, 277.1 [M + Na]$^+$, 293.1 [M + K]$^+$, 531.2 [2M + Na]$^+$.

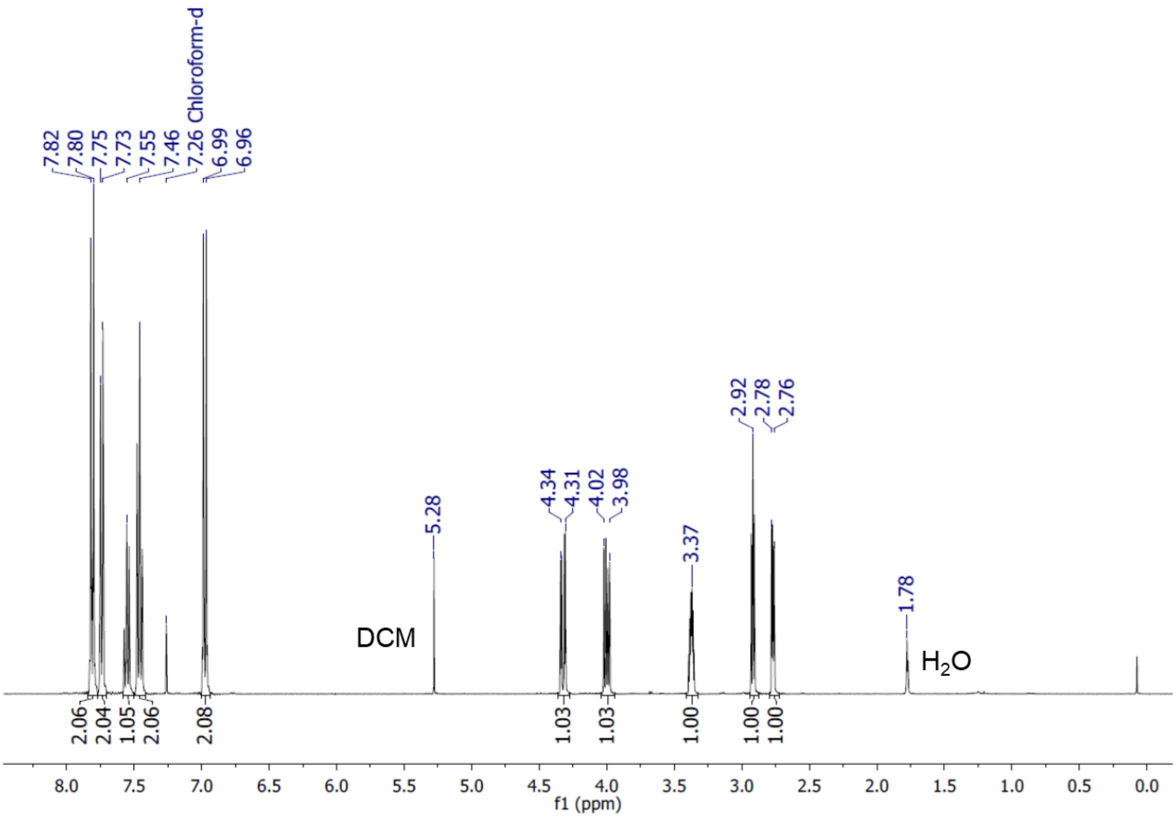

Figure S2. ^{1}H-NMR spectrum of the photo-reactive glycidyl ether comonomer EBP.

2. Block Copolymer Synthesis

<u>General procedure for the MA-AROP:</u>
The initiator $N(Oct)_4Br$ was melted and dried in a flame dried Schlenk flask (100 mL) under high vacuum at 103 °C and dissolved in dry toluene at room temperature. The solution was cooled to 0 °C with an ice bath and the dry monomers GME and EGE were added. The polymerization was initiated via rapid addition of dry Al(*i*-Bu)$_3$ activator solution. After stirring for 15 min at 0 °C, the respective comonomer EBP or AGE was added and the reaction was allowed to proceed for 3 h while warming up to room temperature. The reaction was quenched by adding Milli-Q water (~ 0.5 mL), stirred for 1 h, dried over Na_2SO_4 for 1 h under stirring and filtered. After removing toluene under reduced pressure, the crude polymers were dissolved in Et_2O and residual initiator salts were precipitated by centrifugation at 0 °C. After decanting, Et_2O was evaporated and the block copolymers were dissolved and dialyzed against MeOH for 3 d. PGE block copolymers comprising photo-reactive EBP anchor blocks were protected against light throughout synthesis and workup and stored in stock solutions in ethanol (10 mg mL^{-1}) until further use.

Poly(GME-*ran.*-EGE)-*block*-poly(EBP) (**B**1:3):
$N(Oct)_4Br$ = 120 mg (0.22 mmol, 1 eq), GME = 1.50 mL (16.72 mmol, 76.1 eq), EGE = 5.45 mL (50.15 mmol, 228.2 eq), Al(*i*-Bu)$_3$ = 0.88 mL (0.88 mmol, 4 eq), EBP = 279 mg (1.10 mmol, 5 eq), toluene = 30 mL, yield = 98%; ^1H NMR (500 MHz; $CDCl_3$): δ(ppm) = 7.71 (m, 4H, BP); 7.52-7.43 (m, 3H, BP); 6.92 (m, 2H, BP); 4.19 (m, 1H, $BPOC\underline{H_2}$-); 4.05 (m, 1H, $BPOC\underline{H_2}$-); 3.61-3.45 (m, polymer backbone + $-OC\underline{H_2}CH_3$); 3.33 (s, $-OC\underline{H_3}$); 1.16 (t, $-OCH_2C\underline{H_3}$); ^{13}C NMR [^1H] (126 MHz; $CDCl_3$): δ(ppm) = 197.5 ($\underline{C}$=O); 165.7 ($\underline{C}$(BP)-O-CH_2-polymer backbone); 138.1-115.4 (BP); 78.9-78.6 ($-CH_2\underline{C}H(CH_2OR)O-$ + $BPOC\underline{H_2}$-), 72.8 ($-\underline{C}H_2OCH_3$), 70.6 ($-\underline{C}H_2OCH_2CH_3$), 70.2-69.6 ($-\underline{C}H_2CH(CH_2OR)O-$), 66.7 ($-O\underline{C}H_2CH_3$); 59.2 ($O\underline{C}H_3$); 15.3 ($-OCH_2\underline{C}H_3$); GPC: M_n = 27.1 kg mol^{-1}, PDI = 1.20.

Poly(GME-*ran.*-EGE)-*block*-poly(EBP) (**B**1:1):
$N(Oct)_4Br$ = 116 mg (0.21 mmol, 1 eq), GME = 3.00 mL (33.44 mmol, 157.7 eq), EGE = 3.63 mL (33.44 mmol, 157.7 eq), Al(*i*-Bu)$_3$ = 0.85 mL (0.85 mmol, 4 eq), EBP = 270 mg (1.06 mmol, 5 eq), toluene = 30 mL, yield = 97%; ^1H NMR (500 MHz; $CDCl_3$): δ(ppm) = 7.71 (m, 4H, BP); 7.52-7.43 (m, 3H, BP); 6.92 (m, 2H, BP); 4.19 (m, 1H, $BPOC\underline{H_2}$-); 4.05 (m, 1H, $BPOC\underline{H_2}$-); 3.61-3.45 (m, polymer backbone + $-OC\underline{H_2}CH_3$); 3.33 (s, $-OC\underline{H_3}$); 1.16 (t, $-OCH_2C\underline{H_3}$); ^{13}C NMR [^1H] (126 MHz; $CDCl_3$): δ(ppm) = 197.5 ($\underline{C}$=O); 165.7 ($\underline{C}$(BP)-O-CH_2-polymer backbone); 138.1 115.4 (BP); 78.9-78.6 ($-CH_2\underline{C}H(CH_2OR)O-$ + $BPOC\underline{H_2}$-), 72.8 ($-\underline{C}H_2OCH_3$), 70.6 ($-\underline{C}H_2OCH_2CH_3$), 70.2-69.6 ($-\underline{C}H_2CH(CH_2OR)O-$), 66.7 ($-O\underline{C}H_2CH_3$); 59.2 ($O\underline{C}H_3$); 15.3 ($-OCH_2\underline{C}H_3$); GPC: M_n = 28.4 kg mol^{-1}, PDI = 1.21.

Poly(GME-*ran.*-EGE)-*block*-poly(AGE) (**1a**):
$N(Oct)_4Br$ = 184 mg (0.34 mmol, 1 eq), GME = 2.30 mL (25.63 mmol, 79.1 eq), EGE = 8.40 mL (76.90 mmol, 228.2 eq), Al(*i*-Bu)$_3$ = 1.35 mL (1.35 mmol, 4 eq), AGE = 0.28 mL (2.36 mmol, 7 eq), toluene = 50 mL, yield = 94%; ^1H NMR (500 MHz; $CDCl_3$): δ(ppm) = 5.87 (m, $-OCH_2C\underline{H}CH_2$); 5.27-5.13 (m, $-OCH_2CHC\underline{H_2}$); 3.98-3.97 (m, $-OC\underline{H_2}CHCH_2$); 3.62-3.47 (m, polymer backbone + $-OC\underline{H_2}CH_3$); 3.33 (s, $-OC\underline{H_3}$); 1.17 (t, $-OCH_2C\underline{H_3}$); GPC: M_n = 29.2 kg mol^{-1}, PDI = 1.17.

Poly(GME-*ran.*-EGE)-*block*-poly(AGE) (**2a**):
$N(Oct)_4Br$ = 174 mg (0.32 mmol, 1 eq), GME = 4.50 mL (50.15 mmol, 157.7 eq), EGE = 5.45 mL (50.15 mmol, 157.7 eq), Al(*i*-Bu)$_3$ = 1.27 mL (1.27 mmol, 4 eq), AGE = 0.26 mL (2.23 mmol, 7 eq), toluene = 50 mL, yield = 96%; ^1H NMR (500 MHz; $CDCl_3$): δ(ppm) = 5.87 (m, $-OCH_2C\underline{H}CH_2$); 5.27-5.13 (m, $-OCH_2CHC\underline{H_2}$); 3.98-3.97 (m, $-OC\underline{H_2}CHCH_2$); 3.61-3.47 (m, polymer backbone + $-OC\underline{H_2}CH_3$); 3.33 (s, $-OC\underline{H_3}$); 1.17 (t, $-OCH_2C\underline{H_3}$); GPC: M_n = 30.1 kg mol^{-1}, PDI = 1.15.

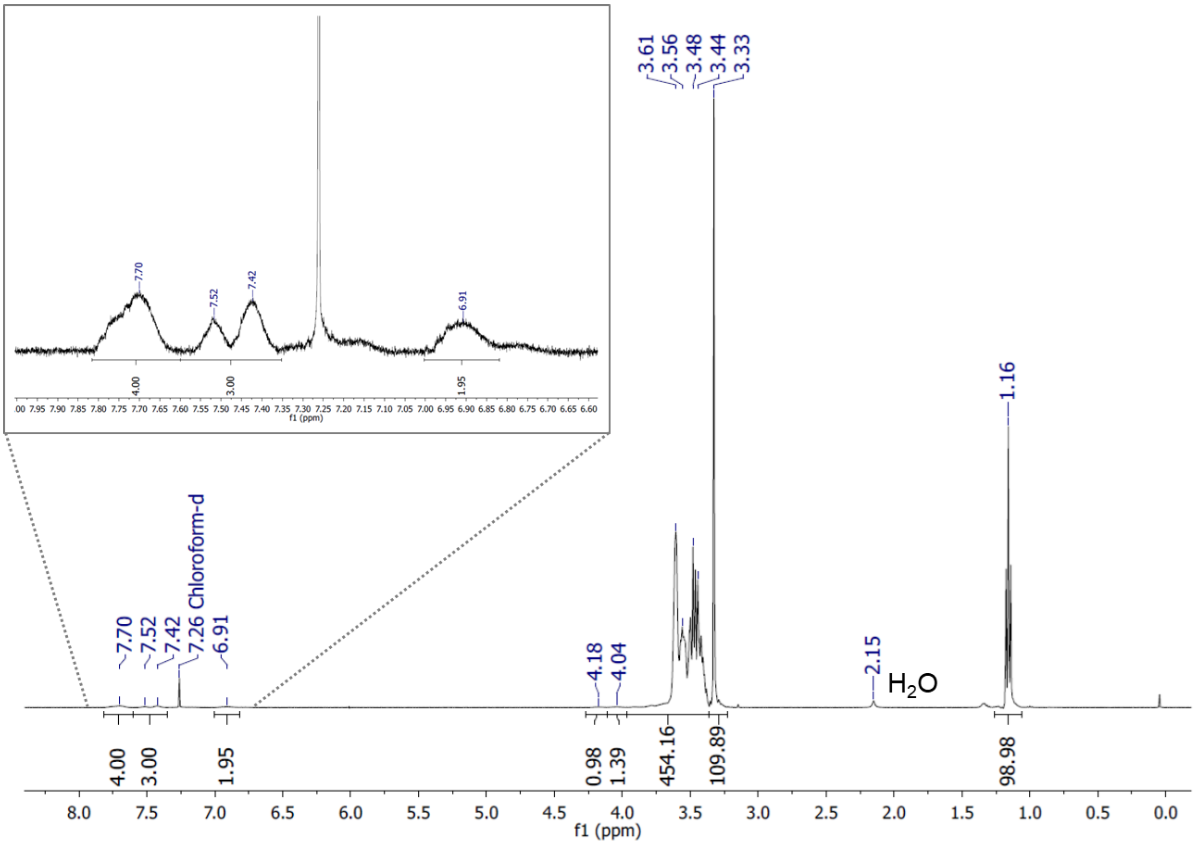

Figure S3. Representative ^{1}H-NMR spectrum of PGE-O-BP block copolymer **B**1:1.

<u>General procedure for PGE post-modification:</u>

PGE block copolymers **1a** and **2a** equipped with short allyl-functional AGE anchor blocks were functionalized with photo-reactive BP units via a two-step post-polymerization protocol.

In the first step, the allyl groups were functionalized with cysteamine groups via thio-ene chemistry based on a previous report by Heinen *et al.* [2]. In brief, PGE block copolymers **1a** or **2a**, Cys-HCl and the photo-initiator DMPA were dissolved in MeOH in a 50 mL reaction vial and purged with Ar for 15 min while protected against light. The reaction mixtures were then irradiated with UV light (Hg lamp, 90 W) for 2 h. The crude products were dialyzed against MeOH for 3 d. After removing the solvent under reduced pressure, the products **1b** and **2b** were obtained as highly viscous pale-yellow solids.

In the second step, the amine group bearing PGE block copolymers were functionalized with BP groups via amide coupling according to a previous report by Yu *et al.* [3]. In brief, to solutions of **1b** or **2b** and 4-CBP in DMF (10 mL) in a 100 mL round bottom flask were added solutions of EDC-HCl in DMF (5 mL). The mixtures were stirred at room temperature for 24 h. The crude product solutions were diluted with MeOH (20 mL) and dialyzed against MeOH for 3 d. After removing the solvent under reduced pressure, **C**1:3 and **C**1:1 were obtained as highly viscous pale-yellow liquids. The polymers were subsequently stored in stock solutions in ethanol (10 mg mL^{-1}) until further use.

Poly(GME-*ran.*-EGE)-*block*-poly(AC) (**1b**):

1a = 2 g (7.1 AGE/chain, 1 eq), Cys-HCl = 269 mg (2.37 mmol, 5 eq), DMPA = 24 mg (0.09 mmol, 0.2 eq), MeOH = 10 mL, conversion (AGE) = 100%, yield = 93%; ^{1}H NMR (500 MHz; $CDCl_3$): δ(ppm) = 3.61-3.42 (m, polymer backbone + $-OC\underline{H_2}CH_3$); 3.33 (s, $-OC\underline{H_3}$); 3.12 (m, $-SCH_2C\underline{H_2}NH_2$); 2.94 (m, $-SC\underline{H_2}CH_2NH_2$); 2.68 (m, $-OCH_2CH_2C\underline{H_2}S-$); 1.85 (m, $-OCH_2C\underline{H_2}CH_2S-$); 1.17 (t, $-OCH_2C\underline{H_3}$).

Poly(GME-*ran.*-EGE)-*block*-poly(AC) (**2b**):
2a = 2 g (6.7 AGE/chain, 1 eq), Cys-HCl = 254 mg (2.24 mmol, 5 eq), DMPA = 23 mg (0.09 mmol, 0.2 eq), MeOH = 10 mL, conversion (AGE) = 100%, yield = 90%; ^{1}H NMR (500 MHz; $CDCl_3$): δ(ppm) = 3.62-3.42 (m, polymer backbone + $-OC\underline{H_2}CH_3$); 3.34 (s, $-OC\underline{H_3}$); 3.12 (m, $-SCH_2C\underline{H_2}NH_2$); 2.94 (m, $-SC\underline{H_2}CH_2NH_2$); 2.68 (m, $-OCH_2CH_2C\underline{H_2}S-$); 1.85 (m, $-OCH_2C\underline{H_2}CH_2S-$); 1.17 (t, $-OCH_2C\underline{H_3}$).

Poly(GME-*ran.*-EGE)-*block*-poly(AC-BP) (**C**1:3):
1b = 1.5 g (5.5 Cys-HCl/chain, 1 eq), 4-CBP = 312 mg (1.38 mmol, 5 eq), EDC-HCl = 265 mg (1.38 mmol, 5 eq), DMF = 15 mL, conversion (Cys-HCl) = 96%, yield = 97%; ^{1}H NMR (500 MHz; $CDCl_3$): δ(ppm) = 7.91 (m, 2H, BP); 7.79-7.75 (m, 4H, BP); 7.58 (m, 1H, BP); 7.46 (m, 2H, BP); 3.61-3.42 (m, polymer backbone + $-OC\underline{H_2}CH_3$); 3.33 (s, $-OC\underline{H_3}$); 2.77 (m, $-SC\underline{H_2}CH_2NHCOBP$); 2.62 (m, $-OCH_2CH_2C\underline{H_2}S-$); 1.82 (m, $-OCH_2C\underline{H_2}CH_2S-$); 1.16 (t, $-OCH_2C\underline{H_3}$); GPC: M_n = 28100 g mol^{-1}, PDI = 1.18.

Poly(GME-*ran.*-EGE)-*block*-poly(AC-BP) (**C**1:1):
2b = 1.5 g (5.6 Cys-HCl/chain, 1 eq), 4-CBP = 318 mg (1.41 mmol, 5 eq), EDC-HCl = 270 mg (1.41 mmol, 5 eq), DMF = 15 mL, conversion (Cys-HCl) = 91%, yield = 96%; ^{1}H NMR (500 MHz; $CDCl_3$): δ(ppm) = 7.91 (m, 2H, BP); 7.79-7.75 (m, 4H, BP); 7.58 (m, 1H, BP); 7.46 (m, 2H, BP); 3.61-3.42 (m, polymer backbone + $-OC\underline{H_2}CH_3$); 3.33 (s, $-OC\underline{H_3}$); 2.77 (m, $-SC\underline{H_2}CH_2NHCOBP$); 2.62 (m, $-OCH_2CH_2C\underline{H_2}S-$); 1.82 (m, $-OCH_2C\underline{H_2}CH_2S-$); 1.16 (t, $-OCH_2C\underline{H_3}$); GPC: M_n = 29800 g mol^{-1}, PDI = 1.20.

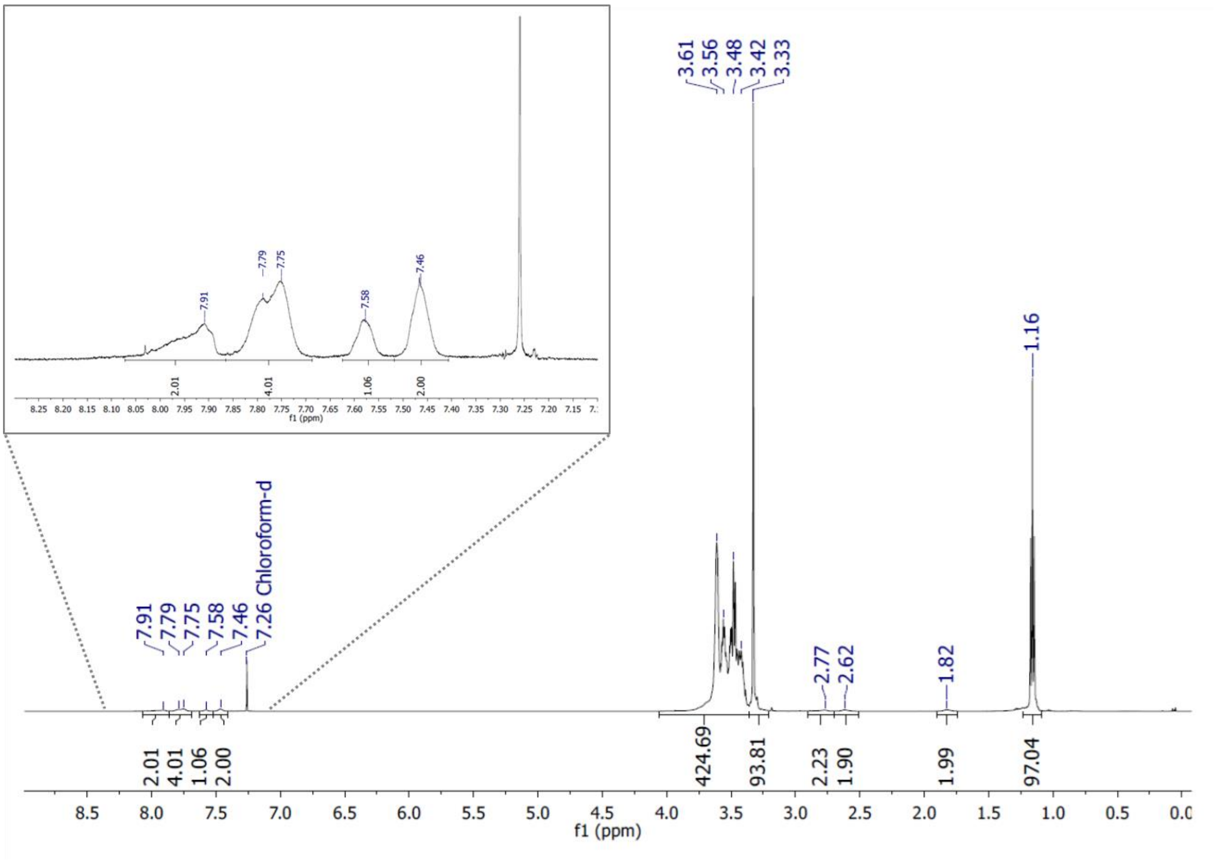

Figure S4. Representative ^{1}H-NMR spectrum of PGE-AC-BP block copolymer **C**1:1.

3. Thermoresponsive Properties of Block Copolymers

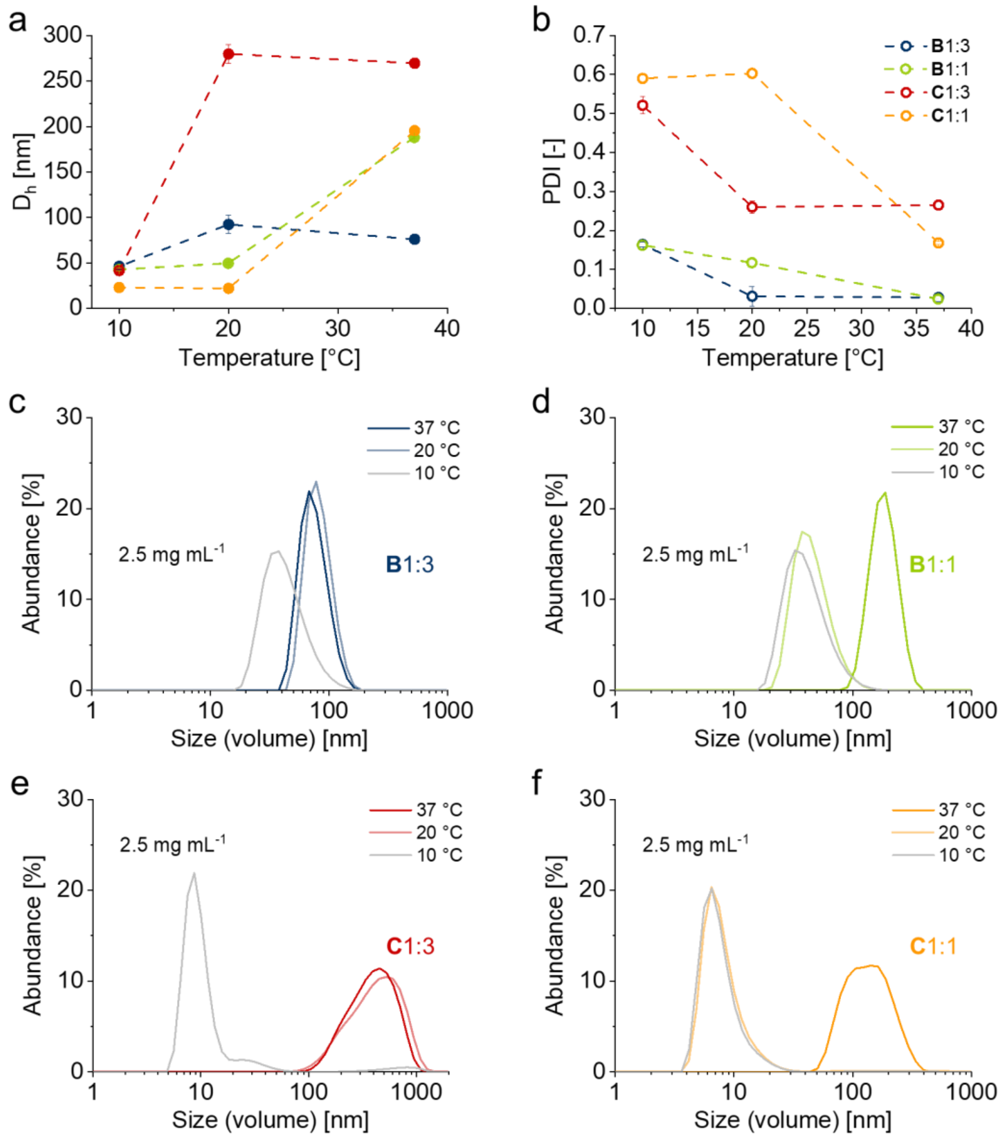

Figure S5. Temperature-dependent volume average hydrodynamic diameter D_h (a), polydispersity PDI (b) and representative particle size distributions of **B**1:3 (c), **B**1:1 (d), **C**1:3 (e) and **C**1:1 (f) in water at 37, 20 and 10 °C and 2.5 mg mL^{-1} determined by DLS. (Error bars indicate SD; n = 6)

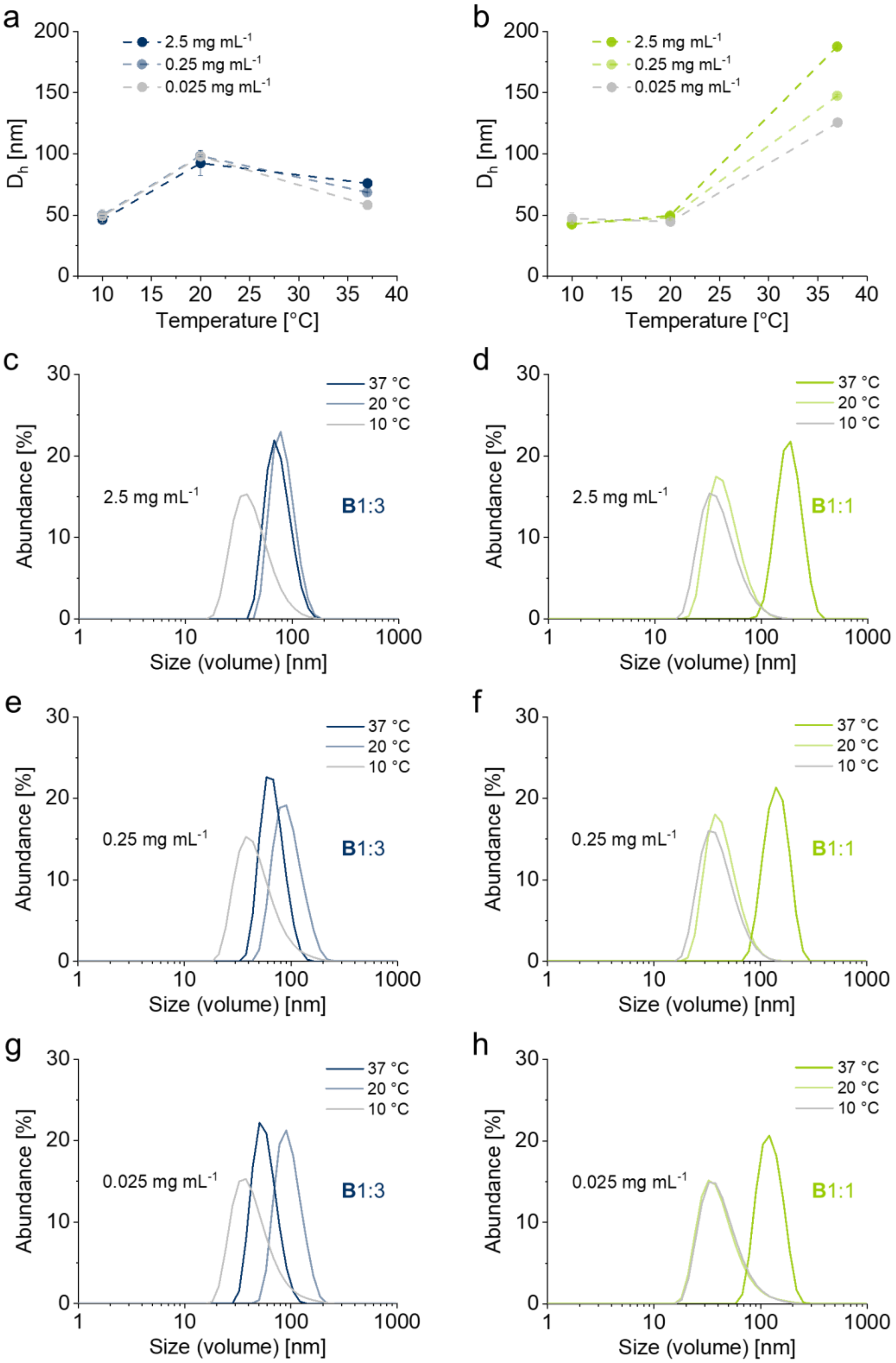

Figure S6. Temperature-dependent volume average hydrodynamic diameter D_h (a, b) and representative particle size distributions of **B**1:3 (c, e, g) and **B**1:1 (d, f, h) in water at 37, 20 and 10 °C and 2.5, 0.25 and 0.025 mg mL^{-1} determined by DLS. (Error bars indicate SD; n = 6)

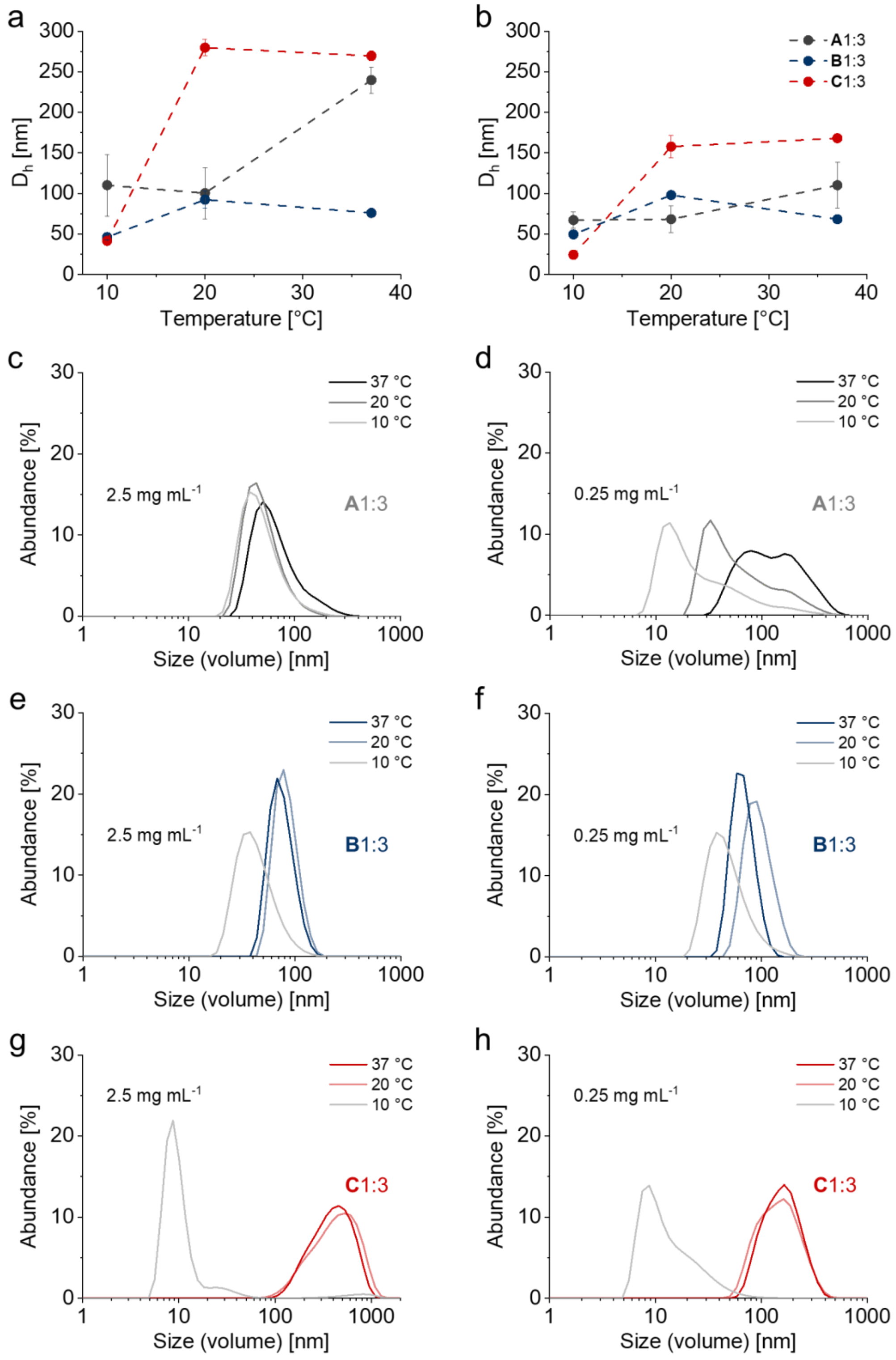

Figure S7. Temperature-dependent volume average hydrodynamic diameter D_h (a, b) and representative particle size distributions of **A**1:3 (c, d), **B**1:3 (e, f) and **C**1:3 (g, h) in water at 37, 20 and 10 °C and 2.5 and 0.25 mg mL^{-1} determined by DLS. (Error bars indicate SD; n = 6)

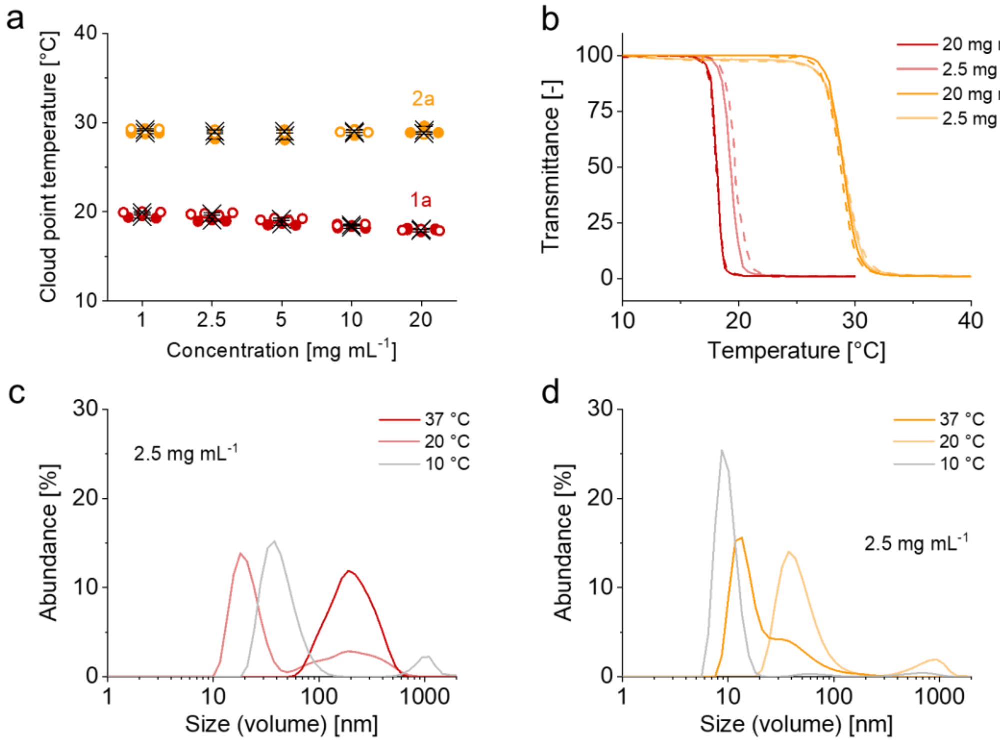

Figure S8. Concentration-dependent CPT (a) from 1 to 20 mg mL^{-1} and representative normalized transmittance curves (b) at 2.5 and 20 mg mL^{-1} of **1a** (red) and **2a** (orange) in water determined by turbidimetry at 500 nm and representative temperature-dependent volume average particle size distributions of **1a** (c) and **2a** (d) in water at 37, 20 and 10 °C and 2.5 mg mL^{-1} determined by DLS. Full dots and continuous lines represent heating cycles. Hollow dots and dashed lines represent cooling cycles. CPTs are plotted for each consecutive heating/cooling cycle together with their mean values (cross) and 90% confidence intervals (whiskers) (n = 4).

4. Self-assembly of Block Copolymer Brushes on Tissue Culture Substrates

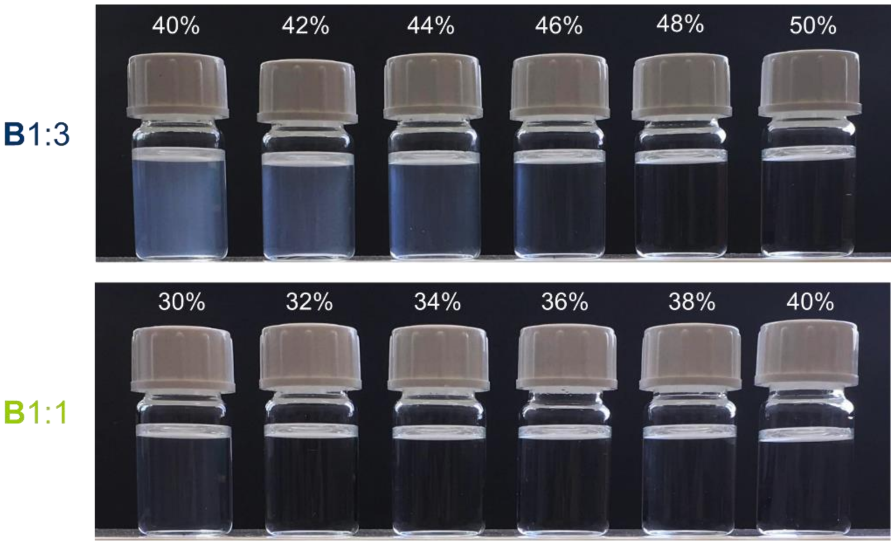

Figure S9. Visual change in turbidity of **B**1:3 and **B**1:1 in water/ethanol mixtures at ethanol concentrations of 40-50 and 30-40% (v/v), respectively, and at a polymer concentration of 0.25 mg mL^{-1}.

To model the theoretical brush conformation under aqueous conditions via the degree of chain overlap $2R_f\ l^{-1}$, we regarded surface-anchored PGE chains as monolayers of spheres on flat tissue culture substrate surfaces [4, 5]. To approximate the degree of chain overlap, we estimated the Flory radius R_f of the PGE chains under bad ($R_f = a\ N^{1/3}$), theta ($R_f = a\ N^{1/2}$) and good solvent ($R_f = a\ N^{3/5}$) conditions using the M_n of the PGE block copolymers determined by GPC and further calculated the anchor distance l and grafting density via the dry thickness of the PGE brushes measured by SE. The grafting densities obtained on PS, PC, PET, TCPS of the block copolymer coatings with a brush conformation are summarized in Figure S10.

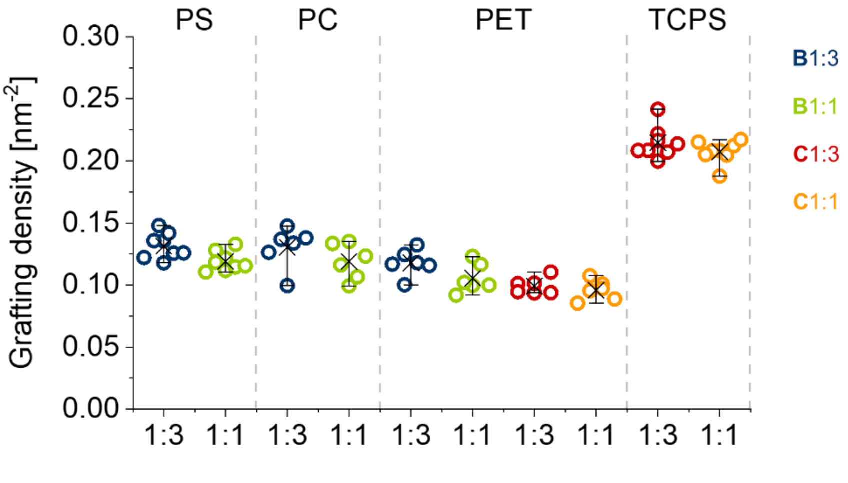

Figure S10. Grafting density of PGE brush coatings **B**1:3 (blue), **B**1:1 (green), **C**1:3 (red) and **C**1:1 (orange) obtained on PS, PC, PET and TCPS via the adsorption/immobilization "grafting-to" process from aqueous/ethanolic solution at a polymer concentration of 0.25 mg mL^{-1}. Grafting densities are plotted for each replicate together with their mean values (cross) and 90% confidence intervals (whiskers) (n = 6).

The theoretically estimated degrees of chain overlap $2R_f\ l^{-1}$ of the PGE coatings are illustrated in Figure S11. We regarded water to be a bad solvent ($R_f = a\ N^{1/3}$) at 37 °C for all brushes. Since the CPTs of **B**1:3/**C**1:3 and **B**1:1/**C**1:1 are in the range of ~ 20 and ~ 30 °C, respectively, we further assumed water to be a theta solvent ($R_f = a\ N^{1/2}$) for **B**1:3 and **C**1:3 and a good solvent ($R_f = a\ N^{3/5}$) for **B**1:1 and **C**1:1.

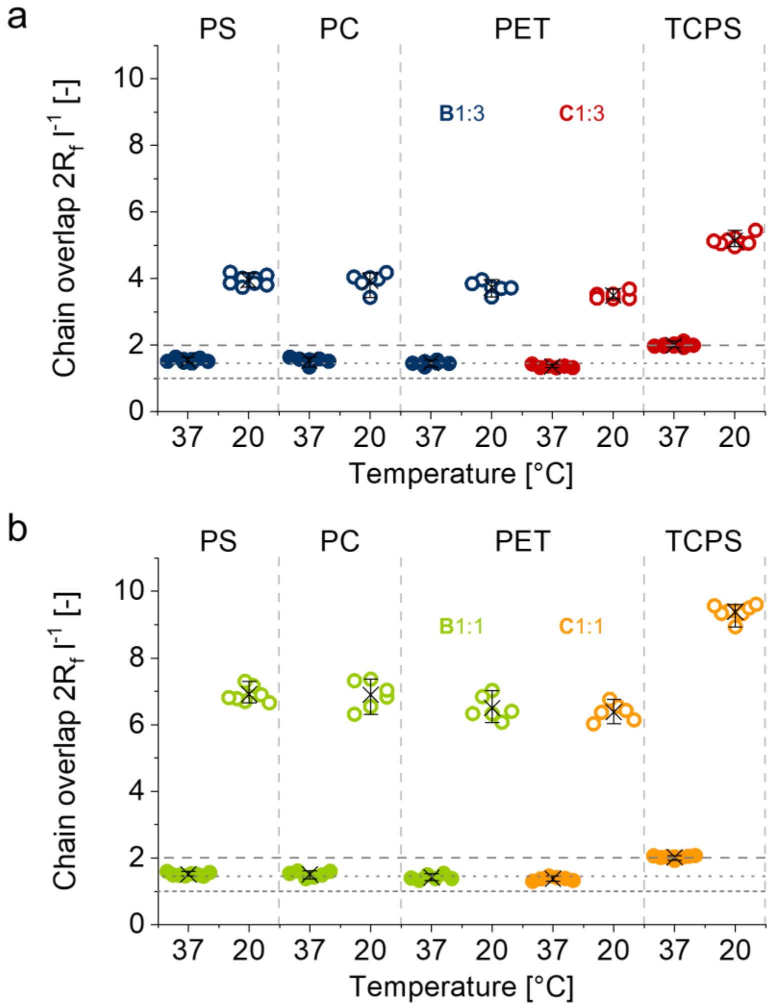

Figure S11. Theoretically estimated, temperature-dependent brush conformation of **B**1:3, **B**1:1, **C**1:3 and **C**1:1 coatings in water at 37 and 20 °C by means of the degree of chain overlap $2R_f\, l^{-1}$. Horizontal dashed and dotted lines represent the start of the chain overlap regime ($2R_f\, l^{-1} \geq 1$), the brush regime at which the substrate surface is completely covered by the polymer coating ($2R_f\, l^{-1} \geq 1.4$) and the extended brush regime ($2R_f\, l^{-1} \geq 2$). Degrees of chain overlap are plotted for each replicate together with their mean values (cross) and 90% confidence intervals (whiskers) (n = 6).

References

1. Jabeen, I.; Pleban, K.; Rinner, U.; Chiba, P.; Ecker, G.F. Structure–Activity Relationships, Ligand Efficiency, and Lipophilic Efficiency Profiles of Benzophenone-Type Inhibitors of the Multidrug Transporter P-Glycoprotein. *J. Med. Chem.* **2012**, *55*, 3261-3273.
2. Heinen, S.; Rackow, S.; Cuellar-Camacho, J.L.; Donskyi, I.S.; Unger, W.E.S.; Weinhart, M. Transfer of functional thermoresponsive poly(glycidyl ether) coatings for cell sheet fabrication from gold to glass surfaces. *J. Mater. Chem. B* **2018**, *6*, 1489-1500.
3. Yu, L.; Hou, Y.; Cheng, C.; Schlaich, C.; Noeske, P.-L.M.; Wei, Q.; Haag, R. High-Antifouling Polymer Brush Coatings on Nonpolar Surfaces via Adsorption-Cross-Linking Strategy. *ACS Appl. Mater. Interfaces* **2017**, *9*, 44281-44292.
4. Heinen, S.; Cuéllar-Camacho, J.L.; Weinhart, M. Thermoresponsive poly(glycidyl ether) brushes on gold: Surface engineering parameters and their implication for cell sheet fabrication. *Acta Biomater.* **2017**, *59*, 117-128.
5. Stöbener, D.D.; Hoppensack, A.; Scholz, J.; Weinhart, M. Endothelial, smooth muscle and fibroblast cell sheet fabrication from self-assembled thermoresponsive poly(glycidyl ether) brushes. *Soft Matter* **2018**, *14*, 8333-8343.
